# Bi-allelic variants in the mitochondrial RNase P subunit PRORP cause mitochondrial tRNA processing defects and pleiotropic multisystem presentations

**DOI:** 10.1016/j.ajhg.2021.10.002

**Published:** 2021-10-28

**Authors:** Irit Hochberg, Leigh A.M. Demain, Julie Richer, Kyle Thompson, Jill E. Urquhart, Alessandro Rea, Waheeda Pagarkar, Agustí Rodríguez-Palmero, Agatha Schlüter, Edgard Verdura, Aurora Pujol, Pilar Quijada-Fraile, Albert Amberger, Andrea J. Deutschmann, Sandra Demetz, Meredith Gillespie, Inna A. Belyantseva, Hugh J. McMillan, Melanie Barzik, Glenda M. Beaman, Reeya Motha, Kah Ying Ng, James O’Sullivan, Simon G. Williams, Sanjeev S. Bhaskar, Isabella R. Lawrence, Emma M. Jenkinson, Jessica L. Zambonin, Zeev Blumenfeld, Sergey Yalonetsky, Stephanie Oerum, Walter Rossmanith, Genomics England Research Consortium, Wyatt W. Yue, Johannes Zschocke, Kevin J. Munro, Brendan J. Battersby, Thomas B. Friedman, Robert W. Taylor, Raymond T. O’Keefe, William G. Newman

**Affiliations:** 1 Institute of Endocrinology, Diabetes, and Metabolism, Rambam Health Care Campus, Haifa 3109601, Israel; 2 Division of Evolution, Infection, and Genomics, School of Biological Sciences, University of Manchester, Manchester M13 9PL, UK; 3 Manchester Centre for Genomic Medicine, St Mary’s Hospital, Manchester University NHS Foundation Trust, Manchester M13 9WL, UK; 4 Department of Genetics, Children’s Hospital of Eastern Ontario, Ottawa, ON K1H 8L1, Canada; 5 Wellcome Centre for Mitochondrial Research, Clinical and Translational Research Institute, Faculty of Medical Sciences, Newcastle University, Newcastle upon Tyne NE2 4HH, UK; 6 Royal National ENT and Eastman Dental Hospital, University College London Hospitals, London WC1E 6DG, UK; 7 Neurometabolic Diseases Laboratory, Bellvitge Biomedical Research Institute, L’Hospitalet de Llobregat, and Center for Biomedical Research on Rare Diseases, 08908 Barcelona, Spain; 8 Paediatric Neurology Unit, Hospital Universitari Germans Trias i Pujol, Universitat Autònoma de Barcelona, 08916 Barcelona, Spain; 9 Catalan Institution for Research and Advanced Studies, 08010 Barcelona, Spain; 10 Unit of Mitochondrial and Inherited Metabolic Diseases, Pediatric Department, University Hospital 12 de Octubre, National Reference Center, European Reference Network for Hereditary Metabolic Disorders, 28041 Madrid, Spain; 11 Institute of Human Genetics, Medical University Innsbruck, Innsbruck 6020, Austria; 12 Laboratory of Molecular Genetics, National Institute on Deafness and Other Communication Disorders, National Institutes of Health, Bethesda, MD 20892-3729, USA; 13 Department of Pediatrics, Children’s Hospital of Eastern Ontario, University of Ottawa, Ottawa, ON K1H 8L1, Canada; 14 The Royal London Hospital, Whitechapel Road, Whitechapel, London E1 1FR, UK; 15 Institute of Biotechnology, University of Helsinki, 00790 Helsinki, Finland; 16 Rappaport Faculty of Medicine, Technion - Israel Institute of Technology, Haifa 3109601, Israel; 17 Department of Pediatric Cardiology, Rambam Health Care Campus, Haifa 3109601, Israel; 18 Newcastle MX Structural Biology Laboratory, Newcastle University, Medical School, NUBI Framlington Place, Newcastle upon Tyne NE2 4HH, UK; 19 Center for Anatomy and Cell Biology, Medical University of Vienna, 1090 Vienna, Austria; 20 Manchester Centre for Audiology and Deafness, School of Health Sciences, University of Manchester, Manchester M13 9PL, UK; 21 Manchester University NHS Foundation Trust, Manchester M13 9WL, UK

**Keywords:** mitochondria, Perrault syndrome, PRORP, sensorineural hearing loss, primary ovarian insufficiency, leukodystrophy, RNase P, rare disease, MRPP3

## Abstract

Human mitochondrial RNase P (mt-RNase P) is responsible for 5′ end processing of mitochondrial precursor tRNAs, a vital step in mitochondrial RNA maturation, and is comprised of three protein subunits: TRMT10C, SDR5C1 (HSD10), and PRORP. Pathogenic variants in *TRMT10C* and *SDR5C1* are associated with distinct recessive or x-linked infantile onset disorders, resulting from defects in mitochondrial RNA processing. We report four unrelated families with multisystem disease associated with bi-allelic variants in *PRORP*, the metallonuclease subunit of mt-RNase P. Affected individuals presented with variable phenotypes comprising sensorineural hearing loss, primary ovarian insufficiency, developmental delay, and brain white matter changes. Fibroblasts from affected individuals in two families demonstrated decreased steady state levels of PRORP, an accumulation of unprocessed mitochondrial transcripts, and decreased steady state levels of mitochondrial-encoded proteins, which were rescued by introduction of the wild-type PRORP cDNA. In mt-tRNA processing assays performed with recombinant mt-RNase P proteins, the disease-associated variants resulted in diminished mitochondrial tRNA processing. Identification of disease-causing variants in *PRORP* indicates that pathogenic variants in all three subunits of mt-RNase P can cause mitochondrial dysfunction, each with distinct pleiotropic clinical presentations.

## Main text

Mitochondrial RNase P (mt-RNase P) is the endonuclease that processes the 5′ end of mitochondrial tRNAs and thereby also releases adjacent mRNAs and rRNAs from the polycistronic primary transcripts.^1^ In humans, the mt-RNase P complex is composed of three proteins, TRMT10C, SDR5C1 (HSD10), and PRORP (called MRPP1, MRPP2, and MRPP3, respectively), each encoded by the nuclear genome.^2^^,^^3^ Bi-allelic variants in *TRMT10C* (MIM: 615423) have been identified in two unrelated individuals with a lethal childhood multisystem disorder, characterized by muscle hypotonia, sensorineural hearing loss (SNHL), metabolic acidosis, and multiple oxidative phosphorylation (OXPHOS) deficiencies (MIM: 616974).^4^ SDR5C1 (also known as HSD10, HADH2, MRPP2, or ABAD [MIM: 300256]), encoded by the X chromosome gene *HSD17B10*, is a moonlighting protein with involvement in multiple biochemical pathways, including isoleucine metabolism.^3^^,^^5^ Pathogenic variants in *HSD17B10* cause HSD10 disease (MIM: 300438), manifesting in males as a severe, infantile-onset neurodegenerative condition with cardiomyopathy.^5^^,^^6^ Both disorders are characterized by defects of mitochondrial tRNA processing.^4^^,^^7^^,^^8^

*PRORP* (previously *KIAA0391* [MIM: 609947]) encodes the endonuclease subunit of the mt-RNase P complex. Here, we describe four families with overlapping phenotypes resulting from bi-allelic variants in *PRORP*. All individuals or their guardians provided written informed consent to participate in the gene discovery study in accordance with local regulations (see supplemental information).

Affected individuals from two families, F1 and F2, presented with SNHL, which was accompanied in the affected females in F1 by primary ovarian insufficiency, consistent with a diagnosis of Perrault syndrome (MIM: 233400).^9^ In family F3, there was childhood onset of SNHL, lactic acidosis, and leukoencephalopathy, whereas affected individuals in family F4 presented with leukoencephalopathy. Recent reports of some individuals with variants in genes associated with Perrault syndrome have expanded the phenotypic spectrum to include presentations with childhood metabolic crises^9^^,^^10^ and leukoencephalopathy.^9^^,^^11^

**Figure 1:**
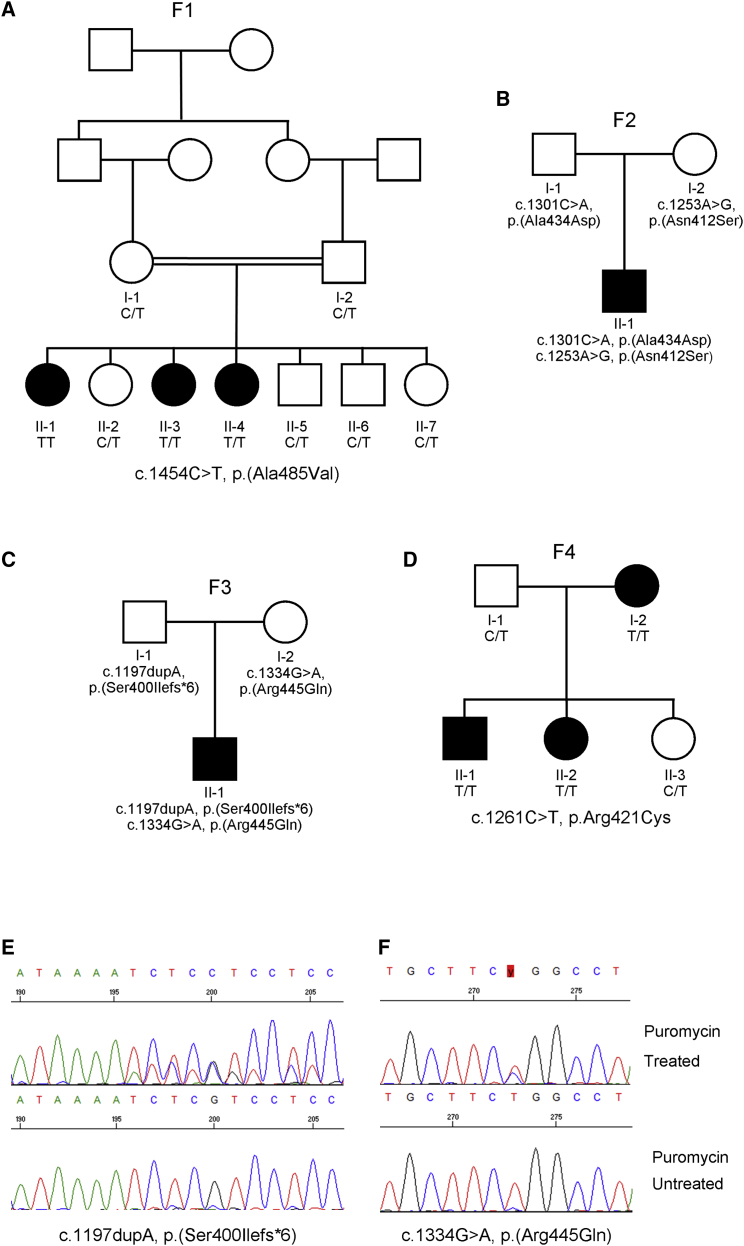
Variants in *PRORP* in four affected families result in pleiotropic clinical presentations (A) The pedigree for a family (F1) with a variant in *PRORP*. (B) The pedigree for the family F2. (C) The pedigree for the family F3 and compound heterozygous variants in *PRORP*. (D) The pedigree for a family (F4) with three affected individuals. Filled symbols indicate affected individuals. (E and F) Sanger sequencing trace for the variants c.1197dupA and c.1334 G>A (highlighted in red) in the proband from F3 (II-1) with cDNA from puromycin treated and untreated fibroblasts. We display sequences in the reverse orientation to prevent masking of the missense variant by the frameshift variant. Sanger sequencing of the cDNA revealed that the frameshift c.1197dupA variant was present in the puromycin-treated cells, but not the untreated cells (E) and the missense variant c.1334G>A was present as a hemizygous change in the untreated samples (F), indicating that the frameshift transcript undergoes nonsense-mediated decay (E and F).

Family 1 (F1) is composed of three affected female siblings, two unaffected female siblings, two unaffected male siblings, and their unaffected parents (Figure 1A). At the last assessment the affected sisters were aged 30, 28, and 26 years of age. All three affected sisters presented with absent middle ear acoustic reflex, despite normal tympanometry, when tested in infancy and subsequent audiology examinations in each sister revealed profound bilateral SNHL (>90 dB hearing level at all frequencies) (Figure S1A). The three affected sisters each presented in their late teenage years with primary amenorrhea, consistent with a diagnosis of Perrault syndrome (see “GeneReviews” in web resources). Pelvic ultrasound noted the absence of ovarian tissue in all three sisters. Hormonal profiles indicated hypergonadotropic hypogonadism (Figure S1C) with otherwise normal endocrine and biochemical tests and a 46, XX karyotype. The affected sisters were prescribed estrogen to induce puberty and are currently maintained on hormone replacement therapy. Each affected sibling has mild non-progressive intellectual disability (brain MR imaging has not been performed). Echocardiography for each of the affected sisters was normal. All other physical and neurological examinations were normal.

A homozygous variant in *PRORP*, c.1454C>T (p.Ala485Val) (GenBank: NM_014672.3), was identified in the affected individuals of F1 via autozygosity mapping and whole-exome sequencing. The variant segregated with the phenotype in the family.

Family 2 (F2) comprises a male proband and his unaffected, unrelated parents (Figure 1B). The proband (F2, II-1) was born at 40 + 4 weeks by emergency caesarean section for fetal tachycardia and meconium stained liqor. Hearing loss in the proband was first noted at 3 years of age but formally diagnosed at 5 years, at which age his brain magnetic resonance imaging (MRI) was normal. He was 9 years at last assessment and had bilateral mild to moderate SNHL (Figure S1B). His speech and language skills are delayed as a result of the hearing loss and he wears bilateral hearing aids. No behavioral or neurological issues have been noted and cardiovascular, respiratory, and abdominal system examinations have been unremarkable. A maternally inherited c.1235A>G (p.Asn412Ser) variant and a paternally inherited c.1301C>A (p.Ala434Asp) variant in *PRORP* were identified in the proband from whole genome sequence data generated through the 100,000 Genomes Project (Figure 1B).^12^

In family 3 (F3), a male proband (F3, II-1) of non-consanguineous unaffected parents (Figure 1C) was born by emergency caesarean section for failure to progress at 41 + 2 weeks gestation. Examination shortly after birth found appendicular hypertonia, more pronounced on the left-hand side, and mild dysmorphism (mild hypertelorism, bilateral epicanthal folds, thin vermilion of the lips, and microretrognathia). At 7 months of age, plasma lactic acid levels were increased at 5.6 mmol/L (reference 0.5–2.5 mmol/L) despite normal levels of plasma amino acids, urine organic acids, acylcarnitines, and free and total carnitine. Urine analysis and serum creatinine were normal. Repeat testing at 24 months revealed plasma lactic acid levels still raised at 3.0 mmol/L. Severe feeding difficulties resulted in the insertion of a gastrostomy tube at 15 months. An electroencephalogram (photic stimulation included) at 19 months revealed no evidence of seizure activity. At 20 months, he had severe global developmental delay, diffuse asymmetric hypertonia, acquired microcephaly (head circumference: 45.7 cm, <2^nd^ percentile), and a mild scoliosis. Brain MRI at 13 months revealed periventricular nodular heterotopias, a dysplastic corpus callosum, diffuse sub-cortical white matter loss, and bilateral connatal cysts (Figure S2). Audiological tests were normal at 18 months, but at 3 years, an auditory brainstem response examination demonstrated evidence of auditory neuropathy spectrum disorder consistent with bilateral SNHL.

Microarray analysis performed on the proband (F3, II-1) detected no copy number variants. Whole-exome sequencing identified bi-allelic variants in *PRORP,* a maternally inherited missense variant c.1334G>A (p.Arg445Gln) (rs777185638) and a paternal frameshift variant c.1197dupA (p.Ser400Ilefs^∗^6) (rs764714439), which was shown to result in nonsense-mediated decay of the transcript (Figures 1E and 1F).

Family F4 is a family comprising three affected individuals, including a brother and sister and their affected mother (Figure 1D). The proband (F4, II-1) was 19 years of age at last assessment. He presented with a psychotic disorder, autistic traits, and learning disability at 7 years. At 8 years, he presented with brief generalized seizures consisting of loss of consciousness and generalized stiffening of the body and extremities. The EEG was normal, but he received treatment with levetiracetam with a good response. Recent physical examination showed obesity and genu and talus valgus. Fundoscopy displayed papillary pallor.

Brain MRI indicated bilateral multiple periventricular and subcortical T2 white matter hyperintense lesions with a posterior predominance that remain unchanged in successive controls (Figure S2). Spectroscopy was normal. Electromyogram and nerve conduction studies revealed no evidence of neuromuscular abnormalities. Ocular and auditory nerve response as assessed by visual evoked potential and auditory brainstem response were normal. Metabolic studies indicated increased lactate/pyruvate ratio with normal plasma lactate, plasma amino acids, and urine organic acids. The proband is currently treated with coenzyme Q10, vitamin B2, vitamin C, carnitine, and arginine.

The affected sister of the proband (F4, II-2) was aged 17 years at last assessment. F4, II-2 presented with intrauterine growth retardation, global developmental delay, and seizures in the first years of life. At the age of 15 years, she presented with tremor in her legs, migraines, and hyperglycemia. Lactate levels were normal. Her hearing is normal as are her electromyogram (EMG) and nerve conduction studies. Brain MRI displayed bilateral multiple periventricular and subcortical T2 white matter hyperintense lesions with a posterior predominance that remain unchanged in successive controls (Figure S2). Spectroscopy was normal. Like her brother, she is treated with coenzyme Q10, vitamin B2, vitamin C, and carnitine.

The mother (F4, I-2) of the proband presented with retrobulbar optic neuritis and tonic pupil (a dilated pupil that responded slowly to light) at 39 years of age. Subsequently, she presented with asthenia, myalgias, memory loss, and frequent headaches. She also had two episodes of left hemiparesis and hypoesthesia that resolved in 15 days without specific treatment and repetitive episodes of lower limb thrombophlebitis. Examination revealed afferent pupillary defect, nasal hemianopsia of the right visual field, and abnormal color perception. She also had distal weakness of the lower limbs, areflexia, and vibratory hypoesthesia of the left side of her body. Her hearing is normal, and she has no evidence of ovarian insufficiency. Routine blood tests and metabolic and thrombophilia studies were all normal. Specific genetic testing for metachromatic leukodystrophy, Krabbe disease, CADASIL, and Leber optic neuropathy were negative. Cerebrospinal fluid (CSF) oligoclonal bands and anti-MOG and anti-NMO antibodies were also negative. Visual evoked potentials displayed abnormal conduction in the right eye. EMG and nerve conduction studies were normal. Brain MRI indicated bilateral multiple periventricular and subcortical T2 white matter hyperintense lesions affecting both hemispheres, corpus callosum, pons, and cerebellum with no contrast enhancement and no changes in successive controls (Figure S2).

Whole-genome sequencing identified a homozygous c.1261C>T (p.Arg421Cys) *PRORP* variant in all three affected individuals. The father (F4, I-1) of the two affected children was a carrier for the variant, and subsequently, the family was confirmed to be consanguineous, consistent with the pseudo-dominant inheritance pattern. There was no evidence of any other putative disease-associated variants in the genome and exome datasets in the four families when the filtering steps were applied (supplemental information).

The altered residues in PRORP identified in the affected individuals from the four families are all highly conserved from vertebrates to fly (Figure S3). All of the variants are either absent from gnomAD^13^ or have a very low minor allele frequency and are not present as homozygous variants. All missense variants were predicted to be deleterious by multiple prediction software (Table 1). Of note, homozygous loss-of-function variants in *PRORP* are absent from publicly available databases and from a consanguineous cohort of >3,200 British Pakistani individuals.^14^

**Table 1:** Analysis of variants in *PRORP* in four families with distinct clinical presentations

**Family**	**F1**	**F2**		**F3**		**F4**
**Family details**						

Family details	three affected female siblings	one affected male		one affected male		two affected siblings and their affected mother
Phenotype	Perrault syndrome	SNHL		developmental delay, SNHL, lactic acidosis, and white matter changes		white matter changes

**Variant details**						

Variant	*PRORP*: c.1454C>T (p.Ala485Val)	*PRORP*: c.1235A>G (p.Asn412Ser)	*PRORP*: c.1301C>A (p.Ala434Asp)	*PRORP*: c.1334G>A (p.Arg445Gln)	*PRORP*: c.1197dupA (p.Ser400IlefsX6)	*PRORP*: c.1261C>T (p.Arg421Cys)
Location	Chr14(GRCh37): g.35739636C>T	Chr14(GRCh37): g.35649943A>G	Chr14(GRCh37): g.35735958C>A	Chr14(GRCh37): g.35735991G>A	Chr14(GRCh37): g.35649905dup	Chr14(GRCh37): g.35649969C>T
dbSNP	not present	rs148259590	rs144536804	rs777185638	rs764714439	rs147065101
Zygosity	homozygous	heterozygous	heterozygous	heterozygous	heterozygous	homozygous
Inheritance	N/A	maternal	paternal	maternal	paternal	N/A
gnomAD MAF (count)	not present	0.0001382 (39 heterozygotes, 0 homozygotes)	0.0008385 (237 heterozygotes, 0 homozygotes)	0.00002475 (7 heterozygotes, 0 homozygotes)	0.00003593 (9 heterozygotes, 0 homozygotes)	0.0001279 (36 heterozygotes, 0 homozygotes)

**Prediction tools**						

SIFT	deleterious (0.0)	deleterious (0.0)	deleterious (0.03)	deleterious (0.0)	N/A	deleterious (0.03)
PolyPhen	probably damaging (1.0)	probably damaging (1.0)	benign (0.443)	probably damaging (1.0)	N/A	probably damaging (1.0)
MutationTaster	disease causing (1.0)	disease causing (1.0)	disease causing (0.811)	disease causing (1.0)	N/A	disease causing (1.0)
VarCards	0.91 (extreme)	0.74(extreme)	0.39	0.83 (extreme)	N/A	0.57 (extreme)
CADD	34	26.4	28.2	34	N/A	35
Conservation	highly conserved	highly conserved	moderately conserved	highly conserved	N/A	highly conserved

**Effect on protein**						

Variant prediction	may distort the active site	may interfere with the shape of the active site	reduce the stability of the protein	directly impact nuclease function	loss of function	this region is disordered in the structure so was not modeled
FOLDX change	reduces stability (ΔΔG 3.97 kcal/mol)	slightly reduces stability (ΔΔG 1.61 kcal/mol)	slightly reduces stability (ΔΔG 1.66 kcal/mol)	slightly reduces stability (ΔΔG 2.32 kcal/mol)	N/A	N/A
tRNA processing (approximate % of wild-type)	82%	13%	86%	25%	N/A	81%

All variants mapped to the *PRORP* transcript GenBank: NM_014672.3. SNHL, sensorineural hearing loss; MAF, minor allele frequency.

Mitochondrial tRNAs (mt-tRNAs) are processed at the 5′ end by mt-RNase P^1^ and at the 3′ end by mt-RNase Z (encoded by *ELAC2* [MIM: 605367]).^15^^,^^16^ This tRNA cleavage also releases most of the RNA species from the polycistronic mitochondrial precursor transcripts according to the mitochondrial tRNA punctuation model.^17^^,^^18^ PRORP, as a subunit of mt-RNase P, catalyzes the Mg^2+^-dependent phosphodiester-bond cleavage of 5′ extensions of mitochondrial tRNAs.^2^^,^^19^ The processing of mitochondrial tRNAs proceeds in a stepwise manner and 5′ cleavage by mt-RNase P precedes tRNA 3′ end processing.^1^^,^^18^

**Figure 2:**
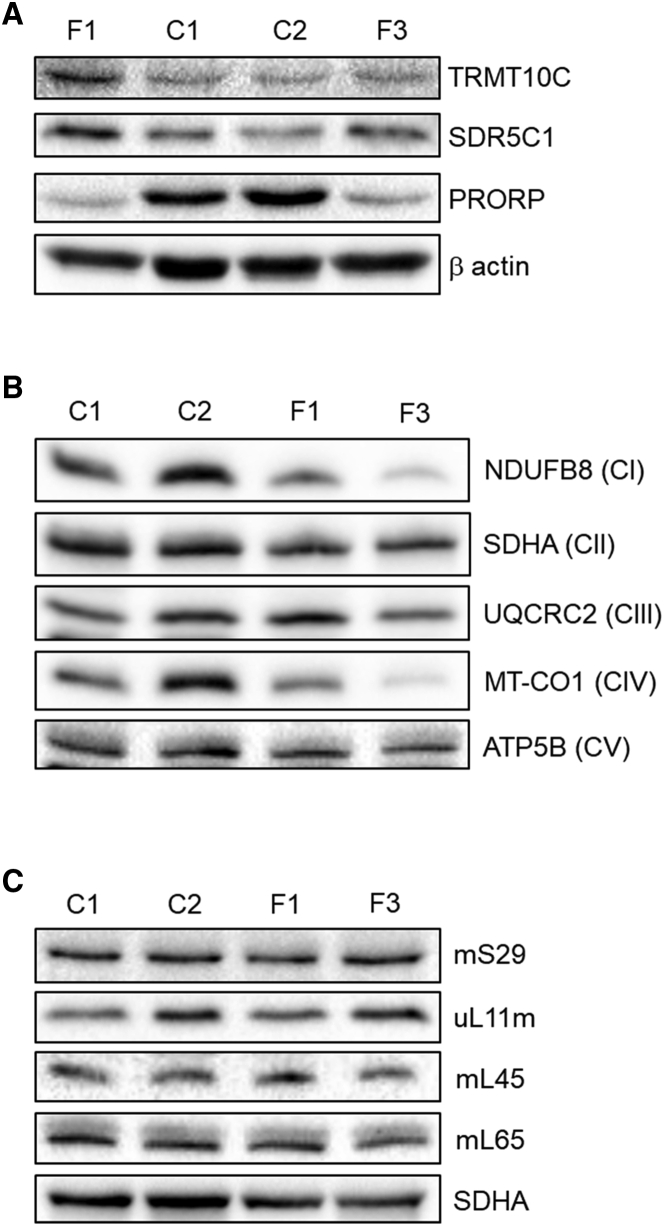
Fibroblasts from affected individuals F1, II-4 and F3, II-1 display reduction in subunits of mt-RNase P and reduced levels of mitochondrial DNA encoded-OXPHOS subunits but no reduction in mitochondrial ribosomal proteins (A) Immunoblot analysis of mt-RNase P subunits TRMT10C, SDR5C1, and PRORP in fibroblasts from two healthy controls (C1 and C2); individual F1, II-4 with the p.Ala485Val variant in PRORP and the individual F3, II-2, who has compound heterozygous variants in *PRORP* (n = 3) (B) Immunoblot analysis of proteins of the five oxidative phosphorylation complexes. Included are two control samples (C1 and C2) and two samples from affected individuals as detailed in (A) (n = 3). (C) Immunoblot analysis of protein subunits of the mitochondrial ribosome. SDHA is included as a loading control. Samples for affected individuals and controls labeled as in (A) (n = 3).

We investigated the steady-state levels of the mt-RNase P subunits TRMT10C, SDR5C1, and PRORP in dermal fibroblasts available from affected individuals in families F1 and F3 by immunoblotting and detected a decrease in PRORP levels in both affected individuals compared to controls (Figure 2A). The decrease in PRORP suggests that the variant p.Ala485Val is either less stable than the wild-type protein or downregulated in affected individuals from family F1. The decreased PRORP levels in F3, II-1 may partially result from absence of protein due to the allele that is subject to nonsense-mediated decay (Figures 1E and 1F). We also detected decreased steady-state levels of respiratory chain complex I (NDUFB8) and complex IV (COXI) subunits in fibroblasts from affected individuals compared to controls—both complexes contain mitochondrial DNA-encoded subunits. There was no change of other OXPHOS components, most notably complex II, which is entirely nuclear encoded (Figure 2B). This profile is consistent with a generalized defect in mitochondrial translation. The decreased stability of complex I and IV subunits was more severe in subject F3, II-1, consistent with his more severe clinical phenotype. There was no noticeable difference in levels of several mitoribosomal proteins (MRPs) between the fibroblasts from affected individuals and controls (Figure 2C), indicating that any effect on translation most likely reflects a defect of transcript processing rather than a defect in the stability or assembly of the mitoribosome itself.

**Figure 3:**
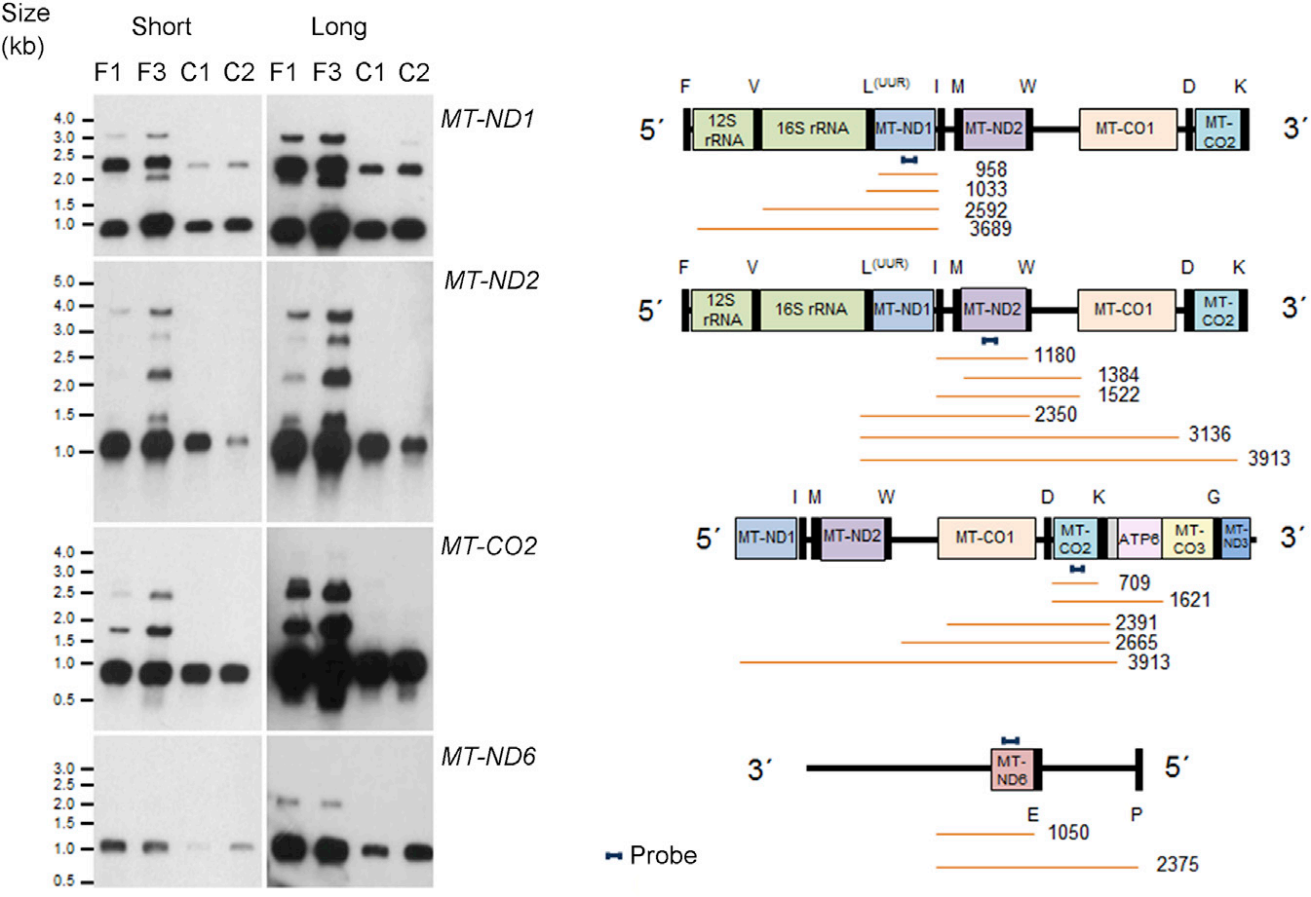
Fibroblasts from affected individuals display impaired mitochondrial RNA processing (A) Northern blot assessment of RNA extracted from F1, II-4 and F3, II-1 fibroblasts and two control (C1 and C2) samples with strand specific probes designed to complement four different mitochondrial gene transcripts: *MT-ND1*, *MT-ND2*, *MT-CO2*, and *MT-ND6*. A long and short exposure of the blots are presented. (B) Schematic representations of mitochondrial genome regions, the probes (red) and expected fragment sizes in bp (orange) are displayed to the right of each blot.

To determine the status of mitochondrial-encoded RNA transcripts in subject dermal fibroblasts, Northern blots were performed. We designed biotinylated strand-specific probes to detect transcripts from four different regions of the mitochondrial genome. An *MT-ND1* probe revealed the accumulation of a precursor RNA of approximately 2.5 kb in the samples from F1, II-4 and F3, II-1 (C1 and C2) (Figure 3), corresponding to unprocessed 16S rRNA-tRNALeu(UUR)-ND1 mRNA and apparently resulting from impaired 5′ end processing of mt-tRNA^Leu(UUR)^. This RNA species was previously termed RNA 19 and observed to be upregulated by the 3243A>G MELAS and other variants in mt-tRNA^Leu(UUR)^.^20^, ^21^, ^22^ A larger RNA species was detected on a longer exposure for the *MT-ND1* probe, indicating that mt-tRNA^Val^ processing is also decreased. The *MT-ND2* and *MT-CO2* probes both detected multiple RNA species seen in affected individuals but not control samples. A *MT-ND6* probe detected a mitochondrial light strand transcript of approximately 2.3 kb in the F1, II-4 and F3, II-1 samples, which can be explained by impaired 5′ processing of mt-tRNA^Glu^ (Figure 3). The presence of multiple large transcripts in the samples from the affected individuals, not seen in the control samples at the same intensity, indicates a deficiency in 5′ processing across multiple mt-tRNA sites.

**Figure 4:**
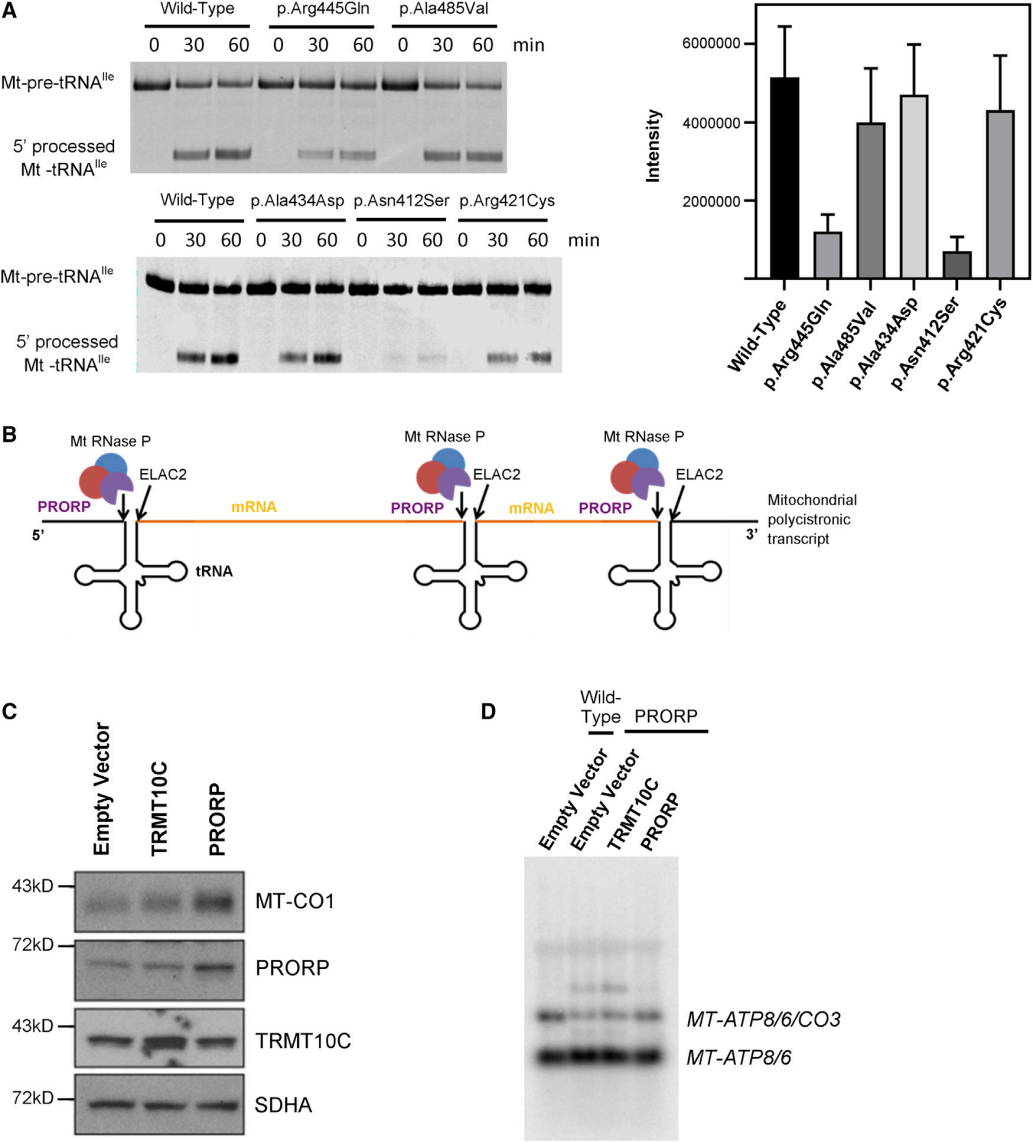
*In vitro* mt-RNase P processing assays reveal all variants produce less 5′-end-processed tRNA than wild-type PRORP; processing defects in subject F3 II-1 are rescued by wild-type *KIAA0391* (A) Mitochondrial pre-tRNA^Ile^ was cleaved by reconstituted recombinant mt-RNase P containing either wild-type or variant *PRORP*, as indicated, resulting in the release of the 5′ leader sequence. Aliquots were taken from the reactions at the time points indicated and resolved by denaturing electrophoresis. Quantitative analysis of pre-tRNA^Ile^ processing revealed an overall decrease in fluorescence intensity of the processed tRNA between wild-type and variants over three replicate experiments. Error bars indicate standard deviation. (B) Cartoon to illustrate the role of PRORP within the RNase P complex in 5′ end cleavage of mitochondrial tRNA transcripts. (C) Immunoblotting of whole cell lysates from fibroblasts stably transduced with the indicated cDNAs. (C) Northern blotting of total RNA hybridized with a strand-specific oligonucleotide probe against *MT-ATP8*.

All disease-associated variants are located in the metallonuclease domain of PRORP (Figure S4A), as revealed by its crystal structure.^23^ Residue Ala485 is situated close to four conserved aspartate residues implicated in metal-ion binding (Figure S4B).^23^ Replacing the conserved alanine at residue 485 with the bulkier valine (F1) could distort the active site and impair catalysis by interfering with proper coordination of the metal ions, thereby reducing the endonucleolytic activity of PRORP. Residue Ala434 is surface exposed and the substitution Ala434Asp (F2) is predicted to slightly reduce the stability of PRORP but no structural change to the protein (Figure S4C). Residue Asn412 is located in the active site next to catalytic residue Asp409 (Figure S4D). Substitution Asn412Ser (also F2) results in no drastic structural changes but may interfere with the shape of the active site, thereby reducing the endonucleolytic activity of PRORP. Residue Arg445 forms stabilizing interactions with essential catalytic residues (e.g., Asp479, Asp478) (Figure S4E). It is likely that Arg445Gln (F3) would directly impact nuclease activity. In 150 orthologs, the residue equivalent to Arg445 is invariably Arg (data not shown). The amino acid at residue 421 is highly variable and is disordered in the structure. During the review of this manuscript, the cryo-EM structure of PRORP in complex with tRNA, TRMT10C, and SDR5C1 was determined.^24^ In addition to supporting the above interpretations of disease-associated variants, this structure reveals that PRORP residue Arg445 forms an interaction with the 5′ end of the tRNA substrate, which will be broken by Arg445Gln (F3). Additionally, Arg421 becomes ordered in the context of the complex and forms stabilizing interactions with residue Glu429, which will be disrupted by Arg421Cys (F4). We investigated whether the disease-associated variants in *PRORP* affected the catalytic activity of the mt-RNase P complex. The three mt-RNase P complex wild-type proteins (TRMT10C, SDR5C1, and PRORP), and the PRORP variant proteins, were individually produced by recombinant expression in bacteria and purified. Recombinant mt-RNase P was reconstituted *in vitro* and 5′ leader processing monitored with fluorescent labeled mt-pre-tRNA^Ile^. All the amino acid variants in PRORP led to a reduction in mt-tRNA^Ile^ cleavage product compared to wild-type PRORP (Figure 4A). The fluorescence intensity of the mt-tRNA^Ile^ cleavage product was quantified. After 30 min from the start of the reaction, the mt-RNase P complexes with variants PRORP p.Arg445Gln (F3) and p.Asn412Ser (F2) displayed the most dramatic decreases in mt-tRNA^Ile^ cleavage products compared to wild-type of approximately 76% and 87%, respectively. The mt-RNase P complexes with variants PRORP p.Ala485Val (F1), p.Ala434Asp (F2), and p.Arg421Cys (F4) reduced 5′ leader processing by approximately 19%, 15%, and 10%, respectively. The reductions in processing persisted after 60 min (Table 1). These data indicate that disease-associated PRORP variants reduce the RNase P activity of the complex *in vitro*. Of note, the greatest reduction of activity is seen in the most severely clinically affected individual (F3, II-1), where a frameshift resulting in loss of function is present in combination with PRORP p.Arg445Gln. However, there are insufficient data to define genotype-phenotype correlations because of the small number of affected individuals ascertained and investigated.

We performed rescue experiments to establish whether expression of wild-type *PRORP* could reduce the accumulation of unprocessed transcripts in the fibroblasts from an affected individual. Fibroblasts from individual F3, II-1 were transduced with a retroviral vector containing the wild-type *PRORP* cDNA or wild-type *TRMT10C* cDNA as a control. Expression of wild-type *PRORP* restored both the amount of PRORP and MT-CO1 protein, whereas TRMT10C and SDHA levels were unaffected (Figure 4B). This result indicates that increased levels of wild-type PRORP in the cells from the affected individual enhances the steady state levels of mitochondrial-encoded MT-CO1 but does not affect levels of nuclear-encoded SDHA. This effect is not seen with the empty vector or *TRMT10C* (Figure 4C). Transducing fibroblasts from the affected individual with *PRORP* also decreased the levels of unprocessed mitochondrial transcripts in the cells to near wild-type levels (Figure 4B). Again, this effect is not seen with the empty vector or the vector containing the coding sequence for *TRMT10C*. Taken together, these data indicate that the expression of wild-type *PRORP* in cells from an affected individual can rescue the molecular defects.

We undertook localization studies of PRORP in the mouse organ of Corti to understand why variants in *PRORP* may be associated with hearing loss (Figure S5). After the onset of hearing, which occurs in mice at postnatal day 12, PRORP is detected around the afferent and efferent synapses of the inner hair cells and the efferent synapses of the outer hair cells, indicating possible importance for synaptic functions after the onset of hearing.

PRORP partially co-localizes with a synaptic marker (SNAP25), indicating that it may not only be present in the mitochondria of efferent synapses but possibly also mitochondria of afferent synapses and nerve fibers around the inner hair cells. The pattern of PRORP staining does not entirely co-localize with mitochondrial marker TOM20. This lack of complete co-localization with TOM20 suggests that the high levels of PRORP found in a subset of mitochondria associated with the synapses and neurons of the organ of Corti hair cells reflect the increased demand for mitochondrial tRNA processing and translation in these cells, which may be a characteristic of a particular type of mitochondria at these locations.^25^, ^26^, ^27^

In summary, we present genetic and functional evidence that bi-allelic variants in *PRORP* are associated with pleiotropic clinical presentations and that *PRORP* should be considered another gene associated with the Perrault syndrome clinical spectrum. Such variability in clinical presentation is not uncommon for mitochondrial disorders and is increasingly being shown for genes associated with Perrault syndrome.^9^ Bi-allelic hypomorphic variants in *CLPP*, for example, are associated with Perrault syndrome,^28^ whereas more deleterious variants result in a more severe phenotype associated with SNHL, seizures, and brain white matter changes.^11^ The clinical spectrum observed in individuals with different bi-allelic *PRORP* variants is consistent with this phenotypic range and most likely reflects altered mitochondrial dysfunction in different tissues at different time points. It is important to note that *PRORP* is ubiquitously expressed in the GTEx dataset. This is consistent with many disorders of mitochondrial function, which have specific clinical phenotypes despite these expression profiles. Notably in the families with multiple affected individuals (F1 and F4), the phenotypes were consistent, indicating that certain *PRORP* variants may result in specific phenotypes.

Similar OXPHOS defects to those seen in individuals with *PRORP* variants have been observed in individuals with pathogenic variants in the mt-RNase P genes *TRMT10C*^4^ and *HSD17B10*,^29^ suggesting a common pathogenic mechanism in these disorders. Despite the similarities in defective mitochondrial tRNA processing, variants in the three subunits of mt-RNase P result in different clinical phenotypes. With our work, we demonstrate that bi-allelic variants in *PRORP* result in mitochondrial dysfunction and that all three subunits of mitochondrial RNase P have now been associated with mitochondrial disease, each with distinct pleiotropic clinical presentations.

## Data and code availability

The *PRORP* variants were submitted to ClinVar (https://www.ncbi.nlm.nih.gov/clinvar/) (GenBank: NM_014672.4; accession numbers SCV001943322–SCV001943327). The exome and genome datasets supporting this study have not been deposited in a public repository because of ethical restriction but are available from the corresponding author on request.

## Web resources

dbSNP, https://www.ncbi.nlm.nih.gov/projects/SNP/
Exome Variant Server, https://evs.gs.washington.edu/EVS/
FoldX, http://foldxsuite.crg.eu/
GenBank, https://www.ncbi.nlm.nih.gov/genbank/
GeneMatcher, https://genematcher.org/
GeneReviews, Newman, W.G., Friedman, T.B., Conway, G.S., and Demain, L.A.M. (2018). Perrault Syndrome, https://www.ncbi.nlm.nih.gov/books/NBK242617/
gnomAD, https://gnomad.broadinstitute.org/
GTEx, https://gtexportal.org/home/
LOVD, https://www.lovd.nl/
MutationTaster, http://www.mutationtaster.org/
OMIM, https://www.omim.org/
PolyPhen-2, http://genetics.bwh.harvard.edu/pph2/
SIFT, https://sift.bii.a-star.edu.sg/

## Supplemental information

Document S1. Figures S1–S5, supplemental material and methods, and supplemental acknowledgments

Document S2. Article plus supplemental information
