# Supplementary material for: Bi-allelic variants in the mitochondrial RNase P subunit PRORP cause mitochondrial tRNA processing defects and pleiotropic multisystem presentations
Source: Am J Hum Genet. 2021 Oct 28;108(11):2195–204. doi: 10.1016/j.ajhg.2021.10.002 (PMC8595931; doi:10.1016/j.ajhg.2021.10.002)
Supplement: Document S1. Figures S1–S5, supplemental material and methods, and supplemental acknowledgments [file mmc1.pdf]

The American Journal of Human Genetics, Volume *108*

Supplemental information

Bi-allelic variants in the mitochondrial RNase P subunit PRORP cause mitochondrial tRNA processing defects and pleiotropic multisystem presentations

Irit Hochberg, Leigh A.M. Demain, Julie Richer, Kyle Thompson, Jill E. Urquhart, Alessandro Rea, Waheeda Pagarkar, Agustí Rodríguez-Palmero, Agatha Schlüter, Edgard Verdura, Aurora Pujol, Pilar Quijada-Fraile, Albert Amberger, Andrea J. Deutschmann, Sandra Demetz, Meredith Gillespie, Inna A. Belyantseva, Hugh J. McMillan, Melanie Barzik, Glenda M. Beaman, Reeya Motha, Kah Ying Ng, James O'Sullivan, Simon G. Williams, Sanjeev S. Bhaskar, Isabella R. Lawrence, Emma M. Jenkinson, Jessica L. Zambonin, Zeev Blumenfeld, Sergey Yalonetsky, Stephanie Oerum, Walter Rossmanith, Genomics England Research Consortium, Wyatt W. Yue, Johannes Zschocke, Kevin J. Munro, Brendan J. Battersby, Thomas B. Friedman, Robert W. Taylor, Raymond T. O'Keefe, and William G. Newman

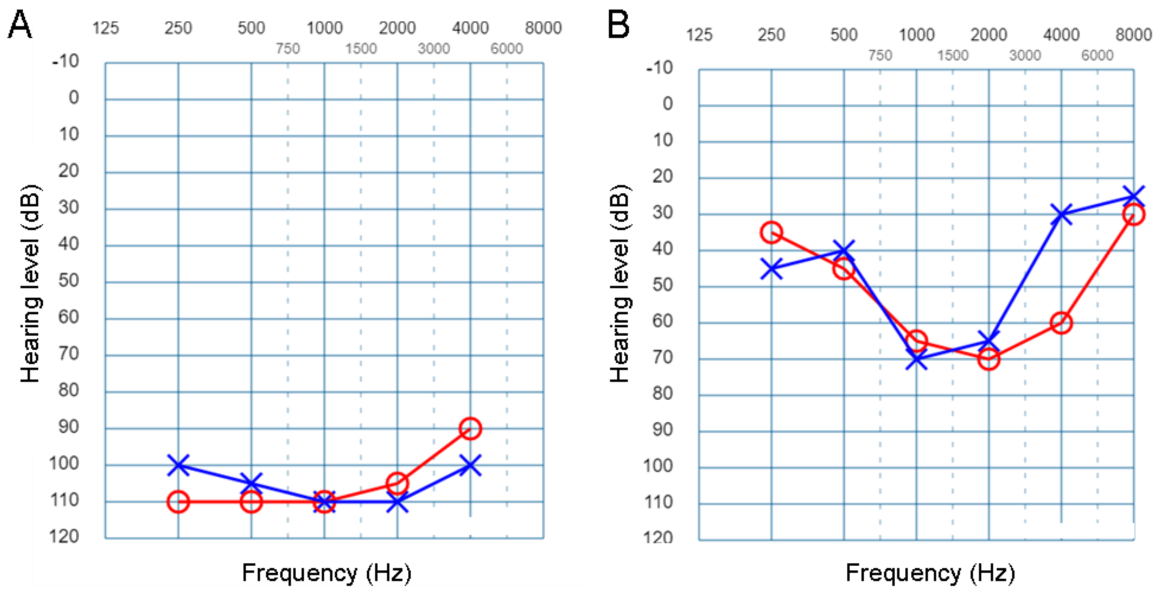

C

	II-1	II-3	II-4	Postmenopausal Reference Values
Follicle Stimulating Hormone (IU/l)	**90.7**	**53.5**	17.7	26.7 - 133.4
Luteinising Hormone (IU/l)	**31.8**	97.9	75.0	10.4 - 64.6
Estradiol (pmol/l)	14.2	**37.3**	12.0	36.7 - 102

Figure S1 – Affected individuals from family F1 and F2 have sensorineural hearing loss, and affected individuals from family F1 have hypergonadotropic hypogonadism
(**A**) Audiogram of affected individual F1 II-4. All three affected sisters show a similar audiometric configuration to F1-II-4, with profound hearing loss across all tested frequencies. (**B**) Audiogram of affected individual F2 II-1. The proband shows bilateral mild to moderate cookie-bite sensorineural hearing loss (SNHL). In both audiograms, the hearing level of the left ear is represented by the blue crosses and the right ear by red circles. The hearing threshold level of a normal adult is 0-20 dB.[1] Audiograms generated using AudGen software. (**C**) Hormone profiles for the three affected sisters in family F1, indicative of hypergonadotropic hypogonadism. Levels of follicle stimulating hormone, luteinising hormone and estrogen in the postmenopausal range are in bold.[2]

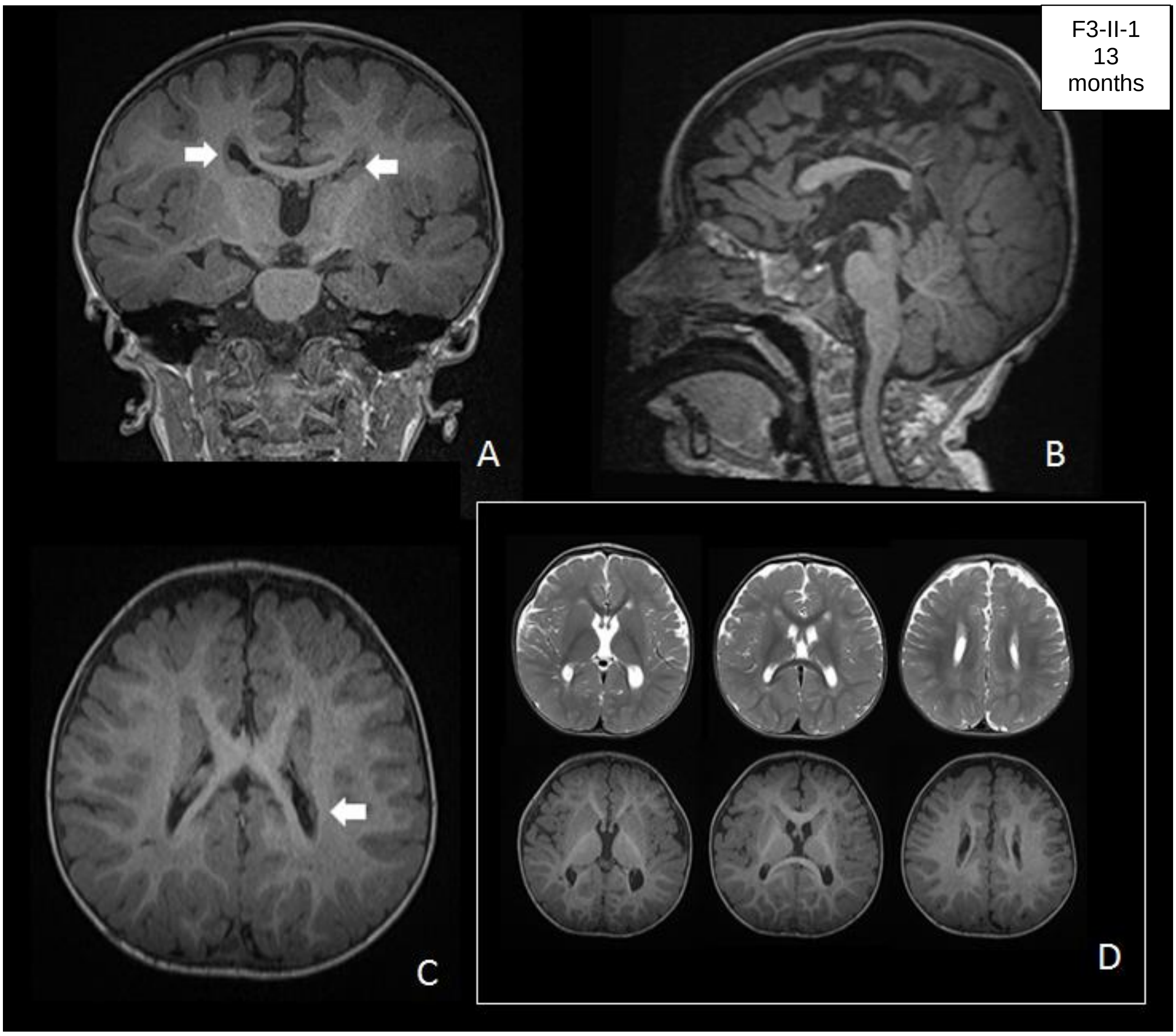
F3-II-1
13
months
A
B
C
D

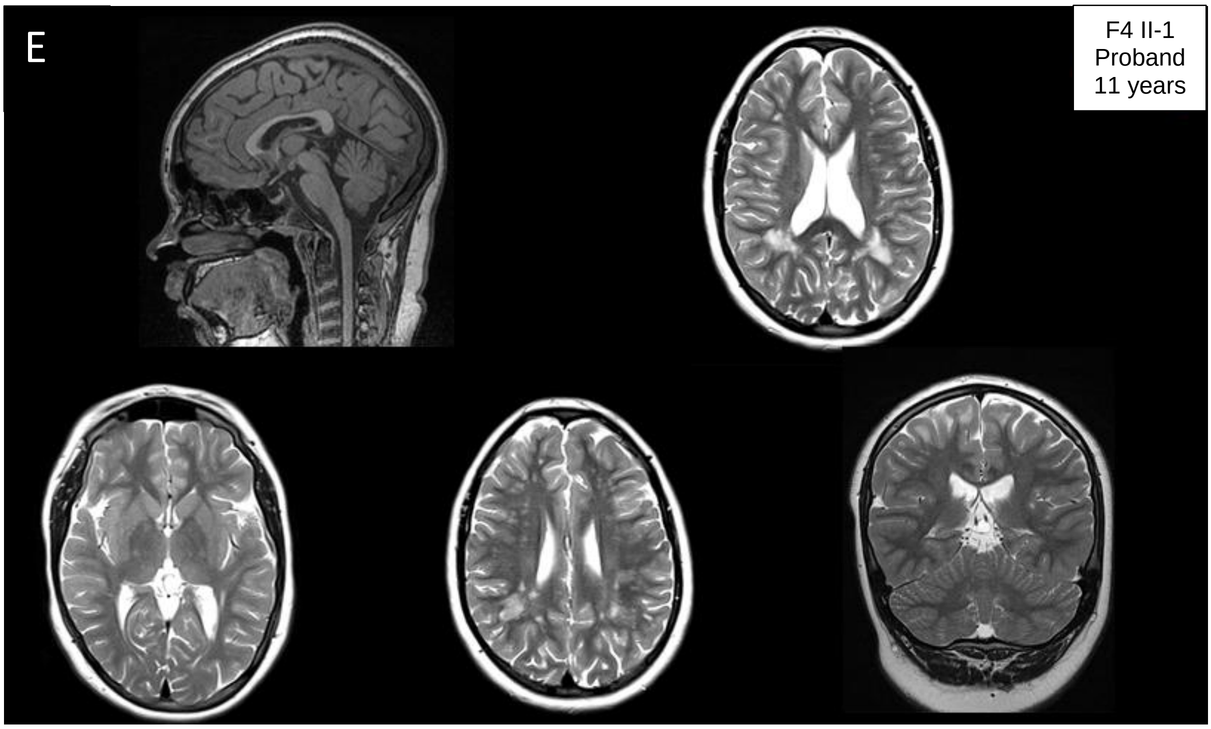
E
F4 II-1
Proband
11 years

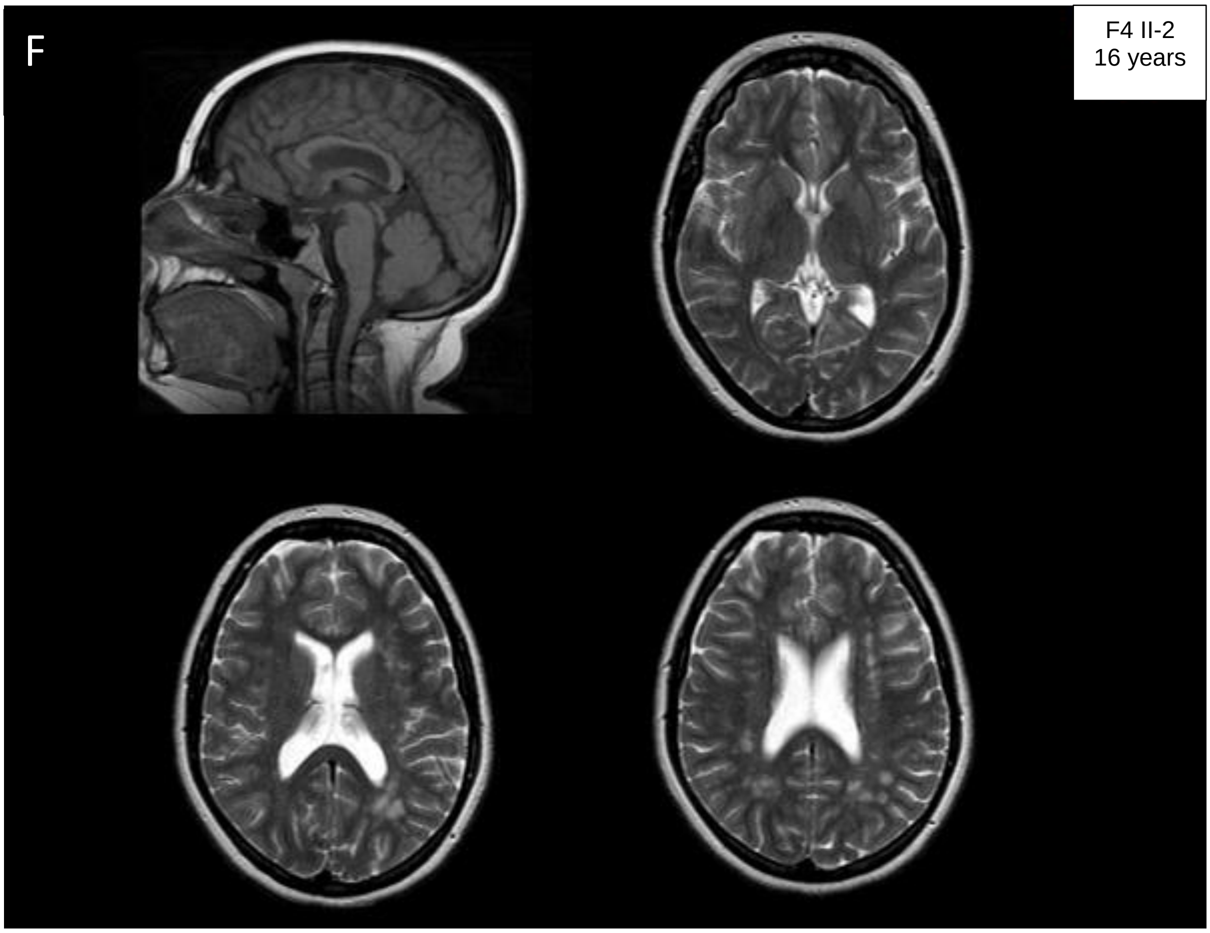
F
F4 II-2
16 years

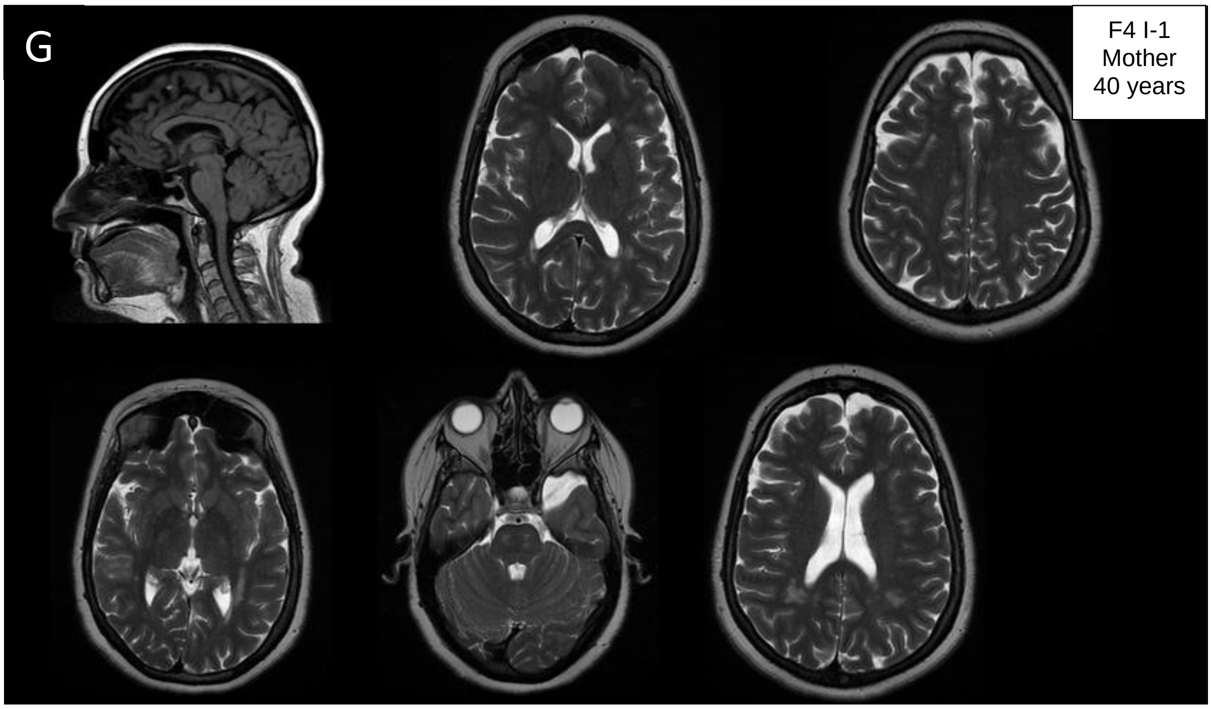

Figure S2 – Brain MRIs for affected individuals in F3 and F4

(A-D) Brain MRI at 13 months of age of individual F3-II-1. Note periventricular cysts – located just above body of lateral ventricles, consistent with connatal cysts (A), dysplastic corpus callosum (B), focal nodular thickening of the posterior horn of left lateral ventricle which may represent a focus of nodular heterotopia (C) and prominent 3rd & lateral ventricles with mild underdevelopment of white matter (D). (F-G) Brain MRI for affected individuals from family F4. Note bilateral multiple periventricular and subcortical T2 white matter hyperintense lesions with a posterior predominance in all affected individuals from this family. The affected mother shows hyper intense lesions involving also the pons (G; bottom right image). For all images age at time of assessment is noted in the white box in the upper right corner of the image.

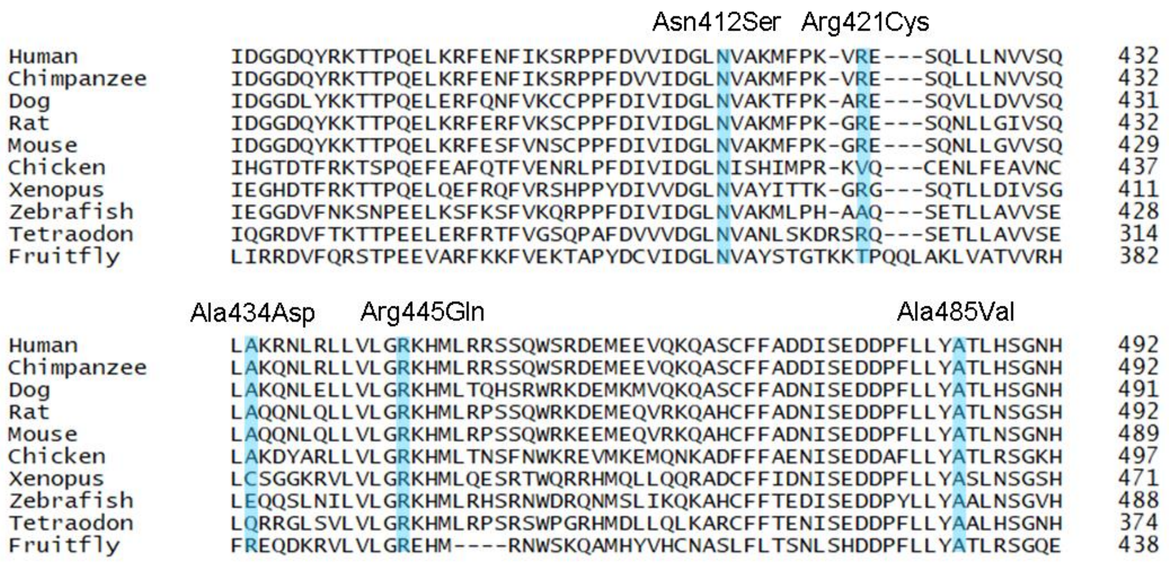

Figure S3 - Conservation of PRORP with variant residues highlighted

The Conservation of PRORP across multiple species. The variant residues in the affected families are highlighted with blue boxes. Numbering relates to the human protein (GenPept: NP_055487.2). The multiple sequence alignments shown use the single letter abbreviations for amino acids while the variants use the three letter HGVS nomenclature. Sequences for each species are as follows; *P. troglodytes* UniProt: H2Q865, *C. lupus familiaris* UniProt: E2RMR1, *R. norvegicus* GenPept: NP_001100200.1, *M. musculus* GenPept: NP_079649.1, *X. tropicalis* UniProt: F6QNJ3, *D.rerio* UniProt: X1WBZ5, *T. nigroviridis* UniProt: H3CE56, *D. melanogaster* GenPept: NP_572309.2.

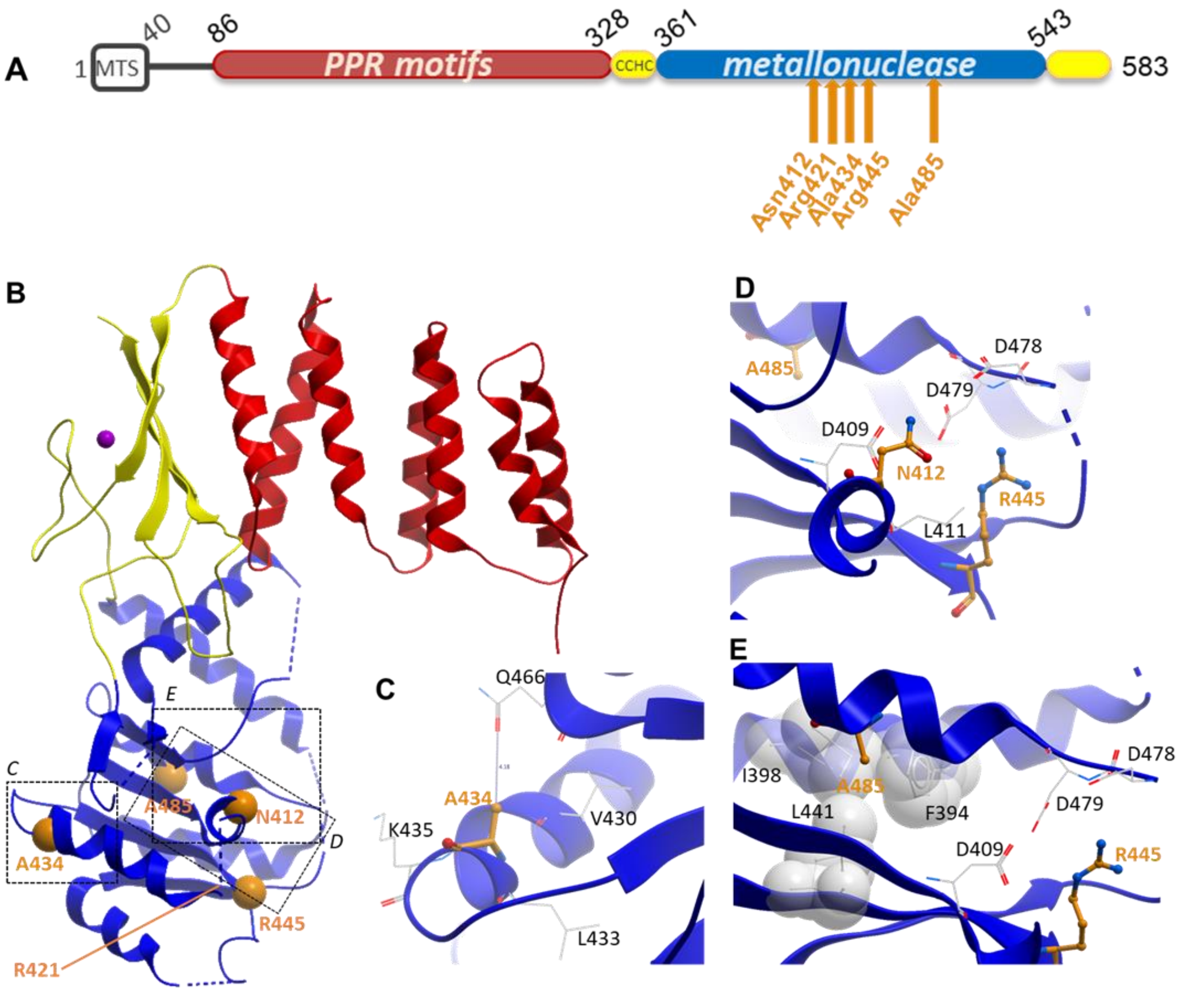

Figure S4 – Localisation of the variant residues in PRORP

(**A**) A schematic domain representation of human PRORP.[3] The location of the variant residues (orange) is noted below the representation. Mitochondrial targeting sequence, MTS; pentatricopeptide repeat domain, PPR. The three letter HGVS nomenclature is used for amino acids (**B**) 3D schematic representation of the protein structure of human PRORP as a ribbon diagram; the enlarged region is part of the metallonuclease domain. (**C, D, E**) The protein structure of human PRORP; the enlarged region is part of the metallonuclease domain. Single letter abbreviations for amino acids are used. The variant amino acids in patients are depicted in orange. Amino acids with interactions with the variant residues are shown in black. The colour of each domain mirrors that of the schematic diagram in A.

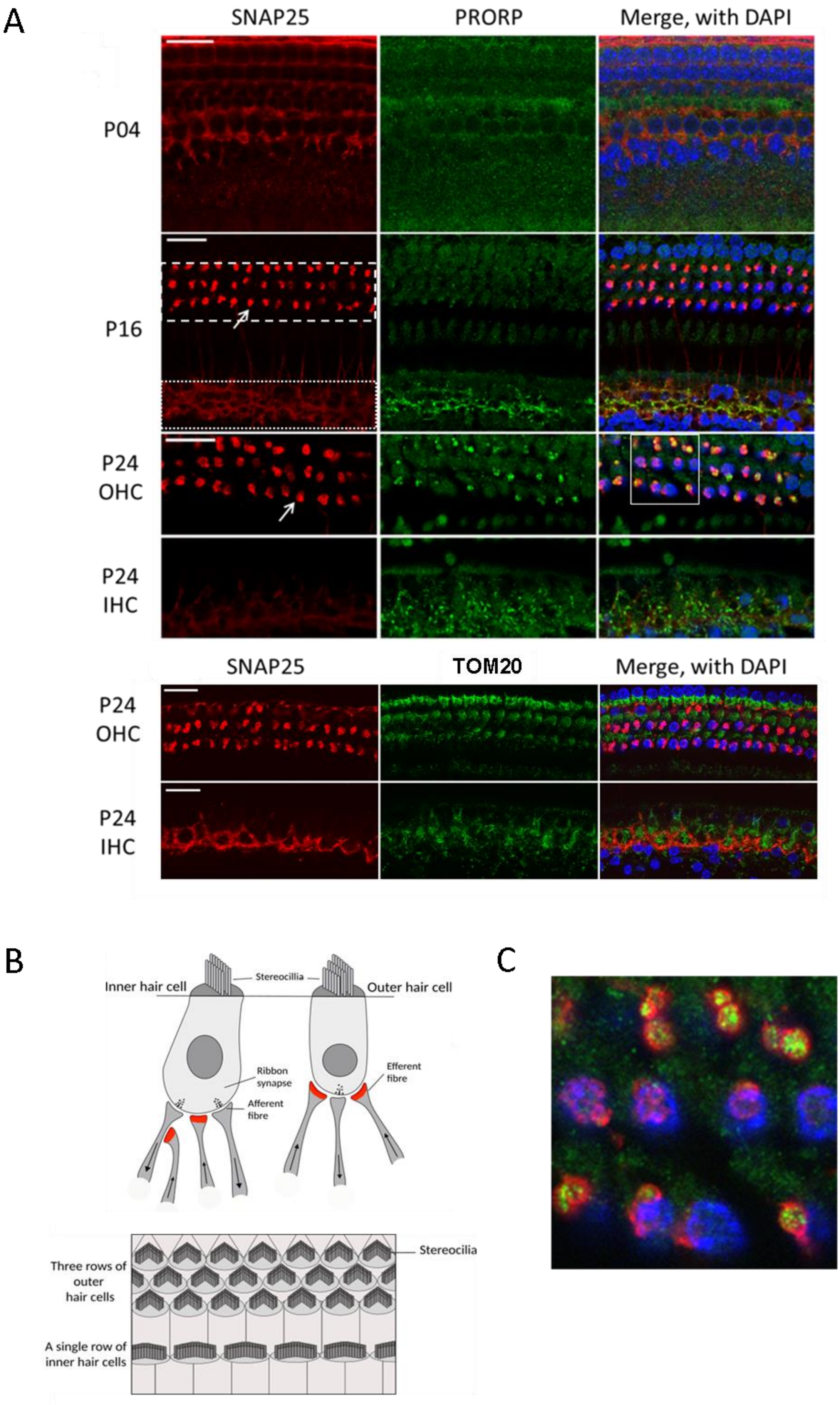
A
SNAP25
PRORP
Merge, with DAPI
P04
P16
P24
OHC
P24
IHC
SNAP25
TOM20
Merge, with DAPI
P24
OHC
P24
IHC
B
Inner hair cell
Stereocilia
Outer hair cell
Ribbon
synapse
Afferent
fibre
Efferent
fibre
Three rows of
outer
hair cells
A single row of
inner hair cells
Stereocilia
C

Figure S5 – Localisation in the mouse organ of Corti reveals high levels of PRORP in the synapses and nerve fibres of hair cells.

(**A**) Confocal fluorescence microscopy optical sections of the whole mount organ of Corti samples from C57/BJ6 mice at postnatal days 4, 16 and 24 (P04, P16 and P24, correspondingly) showing localization of PRORP protein (green). Samples were counterstained with DAPI (nuclear DNA marker, blue) to visualize the nuclei of hair cells and SNAP25 (presynaptic membrane marker, red) to stain efferent synapses at the base of OHCs and nerve fibres and synaptic buttons at the base and around IHCs. Samples at P24 were also stained with TOM20 (mitochondrial marker) as well as SNAP25 and DAPI to show generalized mitochondrial localisation. In the panels for P04, no high level of PRORP immunoreactivity is observed, while at P16, after the onset of hearing, the PRORP signal accumulates in the nerve fibres and at synaptic buttons at the base of IHCs and begins to accumulate at the efferent synaptic buttons of OHCs. The dashed white line outlines the area of the outer hair cell (OHC) efferent synapses, and the dotted white line outlines the area around the inner hair cell (IHC) nuclei and synaptic area. The white arrows point to one of the OHC efferent presynaptic buttons. The P24 panels represent two optical sections through the same organ of Corti sample at different focal plains to visualize OHC synaptic area (top) and IHC synaptic area (bottom). Note that by P24, in the fully mature organ of Corti, the PRORP signal concentrates in both OHC efferent synaptic buttons and in the afferent synaptic buttons of IHCs. The scale bar is 20 µm. (**B**) Schematic representation of the hair cells of the organ of Corti. The top illustration displays the innervation of inner hair cells and outer hair cells during postnatal development. Arrows in the nerve fibres indicate the direction of transmission. The area of SNAP25 staining is shown in red. The lower panel illustration shows the arrangement of hair cells in the organ of Corti in the same orientation as shown in Panel A. (**C)** An enlarged view of the area inside the white box in Panel A showing a concentrated signal of PRORP (green) in the efferent buttons stained by SNAP25 (red); the OHC nuclei shown in blue.

Supplemental Web Resources

AudGen, http://audism.com/audgen

GenBank, https://www.ncbi.nlm.nih.gov/genbank/

UniProt, https://www.uniprot.org/

Materials and Methods

Ethical approval

All individuals or their guardians provided written informed consent in accordance with local regulations. Ethical approval for this study was granted by the National Health Service Ethics Committee (16/WA/0017), University of Manchester; the ethics committee of IDIBELL (CEIC n. PR076/14), and CHEO ethics committee for the Care4Rare Canada Study (ID: 1577), and Genomics England has approval from the HRA Committee East of England – Cambridge South (REC Ref 14/EE/1112).

Autozygosity mapping and whole exome sequencing

Autozygosity mapping was performed on six members of family F1 (II-1, II-2, II-3, II- 4, II-6 and II-7) using the Affymetrix Genome-wide SNP6.0 arrays as previously described.[4] Autozygosity mapping of the array data was performed using the AutoSNPa software.[5] Whole exome sequencing was performed on DNA extracted from lymphocytes from individual F1-II-3. The Agilent SureSelect Human All Exon V5 Panel was used for library preparation and sequencing was performed on the HiSeq 2500 (Illumina) as previously described.[6] Analysis of large deletions and copy number variations was performed on F3-II-1 using Affymetrix Cytoscan HD arrays with analysis via Affymetrix ChAS software (GRCh37/hg19). CNVs were assessed with comparison to databases including the Database of Genomic Variants (DGV). Pathogenic CNVs were identified with reference to databases, including Decipher, ClinVar and ClinGen (ISCA). Whole exome sequencing performed on genomic DNA from all three members of family F3 was performed as a service by GeneDx. Library preparation was performed using the GeneDx propriety technology and sequenced using an Illumina sequencer and using paired-end reads. Reads were aligned to the human genome build GRCh37/hg19 and variants identified using GeneDx software, XomeAnalyzer.

Identification of variants

Autozygosity mapping, performed on six siblings, identified three homozygous regions >2Mb shared between the affected individuals, but not with the unaffected individuals (chromosome 14: 34195478-37228220; chromosome 18: 10090808-12264512; and chromosome 22: 21317876-23416005, Genome build: Hg19). Whole exome sequencing was performed on one affected individual (F1, II-3). After sequence variants in the homozygous regions were filtered to remove variants seen more than once in >800 previously sequenced

exomes, and variants with a minor allele frequency above 1% in dbSNP or the Exome Variant Server (EVS)[7; 8] one variant remained, *PRORP* c.1454C>T; p.(Ala485Val) (Genbank: NM_014672.3).

The variants in family F2 were identified from whole genome sequence data generated through the 100,000 Genomes Project[9] on the parents and affected child and accessed through the dedicated research portal. The trio genome dataset in this family was filtered initially to identify Tier 1 or 2 variants (i.e. rare or known pathogenic variants in genes known to be associated with sensorineural hearing loss). An agnostic approach consistent with an ultra-rare minor allele frequency ($<10^{-5}$) and recessive inheritance pattern revealed maternally inherited *PRORP* c.1235A>G, p.(Asn412Ser) and paternally inherited c.1301C>A, p.(Ala434Asp) variants.

For F3 whole exome sequencing of the family trio was undertaken by GeneDx. Exonic and flanking splice junctions were captured using a proprietary system developed by GeneDx and sequenced on an Illumina platform with 100bp or greater paired end reads. Reads were aligned to human genome build GRCh37/UCSC hg19, and analysed for sequence variants using a custom-developed analysis tool (Xome Analyzer). Mean depth of coverage 94X, quality threshold 98.7%. All available data files were run through the most recent iteration of our previously described bioinformatics pipeline.[10] Family-based reanalysis was completed at the Children's Hospital of Eastern Ontario in collaboration with the referring clinical care team which included (Care4Rare study team members, including the local referring clinician, a clinical geneticist, a laboratory geneticist, genetic counsellor, and/or a post-doctoral fellow). Biallelic variants were identified in *PRORP*, a maternally inherited c.1334G>A p.(Arg445Gln) (rs777185638) and a paternal frameshift variant c.1197dupA p.(Ser400IlefsX6) (rs764714439) were identified.

For F4 genomic DNA was extracted from peripheral blood using standard methods. For WGS, a PCR-free library with 150-bp paired-end read sequences was generated on a HiSeq 2000-4000 platform (Illumina, Inc. USA) at Centre Nacional d'Anàlisi Genòmica (CNAG Barcelona, Spain). Sequences were aligned to hg19 by Burrows-Wheeler Aligner (BWA mem), and single nucleotide variants and small insertions/deletions (indels) were identified using GATK, applying GATK's best practices for germline SNP & indel discovery and annotated by ANNOVAR software. Copy number variants (CNVs) were analyzed by the R package ExomeDepth that uses read-depth data from targeted sequencing experiments and filtered with the Database of Genomic Variants that provides a comprehensive summary of structural variation in the human genome. A recessive model was applied and ultra-rare variants at a minor allele frequency of $<10^{-5}$ and CADD score >20 were filtered. The one candidate variant *PRORP* c.1261C>T, p.Arg421Cys was validated and tested for co-segregation in all family members by Sanger sequencing.

There was no evidence of any other putative disease associated variants in the genome and exome datasets in the four families.

Confirmation of variants

Variants were confirmed in family F1 via Sanger sequencing using the ABI big Dye v3.1 (ThermoFisher) sequencing technology. Primers used were *PRORP*_exon7_ FWD (5'ACACTGTCCTCTGCCTCTTC3') and *PRORP*_ exon7_REV (5'TCTAGGACCTGGCTAGTTCC3')

***PRORP t*ranscript analysis**

Dermal fibroblasts from individual F3, II-1 were grown under standard conditions either in the presence of puromycin (200µg/ml) or without puromycin treatment. RNA was extracted using the Qiagen MiniprepRNA kit and RNA samples were DNAse I treated on column. RNA was converted to cDNA using the Applied Biosystems RNA to cDNA kit. Samples were PCR amplified using primers that cross exon-exon borders and Sanger sequenced using ABI big Dye v3.1 (ThermoFisher) sequencing technology. Primers used were *PRORP*_cDNA_Exon 5_Fwd (5'CATGGTTTGAGAGTGTTCCTGG3') and *PRORP*_cDNA_Exon 5 (5'CTGAGAATACGCTGAAAGGTTAG3').

Assessment of protein and RNA levels in fibroblasts

Western blots for the subunits of mt-RNase P, respiratory chain complexes and mitoribosomal proteins (n=3 for each experiment) were performed using fibroblast cell lysates (F1 II-4 and F3 II-1). Cell lysates were incubated with sample dissociation buffer, separated by 12% SDS–PAGE and immobilized by wet transfer on to PVDF membrane (Immobilon-P, Millipore Corporation). Proteins of interest were bound by overnight incubation at 4°C with primary antibodies followed by HRP-conjugated secondary antibodies (Dako Cytomation) and visualized using ECL-prime (GE Healthcare) and BioRad ChemiDoc MP with Image Lab software. Antibodies used are as follows; TRMT10C (Sigma HPA036671), SDR5C1 (Sigma HPA001432), PRORP (Abcam ab185941), SDHA (Abcam ab14715), NDUFB8 (Abcam ab110242), MT-CO1 (ab14705), UQCRC2 (Abcam 14745), ATP5B (Abcam 14730), MT-CO1 (ab14705), and GAPDH (Abcam 8245) followed by HRP-conjugated secondary antibodies (Dako Cytomation).
Northern blot analysis was performed as previously described.[11] The NorthernMax kit from Ambion was used. Equal amounts of total RNA (2–5 µg range) from fibroblasts was separated on a 1% denaturing agarose gel. RNA was then transferred to nylon membrane (Hybond-N + Amersham, GE Healthcare) by capillary transfer, UV cross-linked and subjected to hybridization with biotinylated probes. Signals were detected using the BrightStar BioDetect kit (Ambion). A biotinylated RNA size marker (BrightStar RNA Millenium Marker, Ambion) was used to determine the size of RNA species. Probe sequences as previously described.[11]

Preparation of the PRORP variant sequences for bacterial expression

The plasmid pET28-b(+) containing the coding sequence for PRORP (MRPP3)[12] was mutagenized as previously described[13] with the synthetic oligonucleotides PRORP_p.A485V (5' GGAGTGCAGTGTG**A**CATACAGAAGGAATGG 3'),PRORP_p.R445Q (5'

CGTCTTAGCATGTGCTTC**_T_**GGCCTAGGACCAGCAGTCG 3'), PRORP_p.N412S (5' GGAAACATTTTGGCAACA**_C_**TGAGACCATCAATGACAAC 3'), PRORP_p.A434N (5' CAGTCGCAGATTCCGTTTG**_T_**CTAGTTGAGAGACGACATTC 3') and PRORP_p.R421C (5' CGACATTCAAGAGAAGTTGAGATTCAC**_A_**AACTTTAGGAAACATTTTGGC 3') with the base altered from wild-type bold and underlined. The potential mutagenized plasmids were extracted using the GenElute HP Plasmid miniprep Kit (Sigma Aldrich) and the variant was confirmed by DNA sequencing.

Recombinant expression and purification of TRMT10C, SDR5C1 and PRORP

PRORP and PRORP variants, as described above, were expressed in *E.coli* Rosetta2 DE3 (Novagen) using Overnight Express TB medium (Novagen). Affinity chromatography of the His-tagged proteins was performed as previously described.[12] Purity was assessed by SDS-PAGE. Aliquots of purified proteins were dialysed overnight at 4°C in 20 mM Tris-Cl pH 7.4, 100 mM NaCl, 15% glycerol, then flash frozen and stored at -80°C. *TRMT10C* in pET28-b(+)[12] was subcloned into pET21d and co-expressed with SDR5C1 in pET28-b(+)[12] in *E.coli* Rosetta2 DE3 using Overnight Express TB medium at 19°C. Purified proteins were dialysed overnight at 4°C in 20 mM Tris-Cl pH 8, 200 mM NaCl, 2mM DTT, 15% glycerol, then flash frozen and stored at -80°C.

Preparation of mitochondrial pre-tRNA transcripts

The template for pre-$tRNA^{Ile}$ (phI2) was as described previously.[12] *In vitro* transcription was carried out with the T7 RiboMax Express system (Promega) according to the manufacturer's instructions with phI2 linearized with XbaI and 2.5µM aminoallyl-UTP-ATTO-680 (Jena Biosciences). RNA was purified by ethanol precipitation.

Pre-tRNA processing assays

Pre-tRNA processing assays were performed as previously described.[12,14] 6% (w/v) acrylamide 8M urea gels were used to resolve ATTO-680 labelled mt-tRNA substrate and cleavage products. Gels were visualised using the LI-COR Odyssey CLx imaging system and band quantitation carried out using the Image Studio software. Aliquots of the tRNA processing reactions were taken at the start of the reaction (0 minutes), after 30 minutes and 60 minutes from the start in three independent assays. Visible processed tRNA bands at time-points 30 minutes and 60 minutes were measured as a proxy for mt-RNase P activity. The relative intensities of tRNA processed by mt-RNase P with wild-type and PRORP variants from three independent assays were quantitated at 30 minutes and presented with standard deviation.

Rescue experiments

RNA isolation and northern blotting

Total RNA from cultured cells was isolated with the Monarch Total RNA Miniprep kit (NEB) according to the manufacturer's instructions. For northern blotting, 5 µg of total RNA

from each sample was separated through a 1.2% agarose-formaldehyde gel and transferred to HybondTM-N+ membrane (GE Healthcare) by neutral transfer. Using T4 Polynucleotide Kinase (NEB) and ATP (γ-^{32}P), an oligonucleotides probe (MT-ATP8, 5'-TGGGTGATGAGGAATAGTGTAAGGAG) was radiolabeled for hybridisation (25% Formamide, 7% SDS, 1% BSA, 0.25M sodium phosphate pH 7.2, 1mM EDTA pH 8.0, 0.25M NaCl2) overnight at 37°C. Membranes were washed first with 2× SSC/0.1% SDS for 60 min, followed by 0.5× SSC/0.1% SDS for 60 min and finally in 0.1× SSC/0.1% SDS for 30 min. All washings were performed at 37°C. The membranes were dried, exposed to a Phosphoscreen (GE Healthcare) and scanned with Typhoon 9400 (GE Healthcare).

Retroviral expression

Full-length cDNAs of human TRMT10C and PRORP were generated by reverse transcription using Superscript IV (Invitrogen) with an oligo dT primer from total RNA isolated from wild-type cultured fibroblasts followed by PCR using specific primers with KAPA HiFi (Sigma-Aldrich) for Gateway cloning into pDONR201. Both cDNAs were verified by Sanger sequencing and sub-cloned into a Gateway-converted pBABE-puromycin retroviral vector. Retrovirus was generated following transfection of plasmids into the Phoenix packaging cell line, followed by transduction into immortalized wild-type and fibroblasts from an affected individual (F3, II-1). Transduced cells were selected with puromycin to select for stable cultures.

Immunoblotting

Cells were lysed in phosphate buffered saline, 1% dodecyl-maltoside (DDM), 1 mM PMSF (phenylmethylsulfonyl fluoride), and complete protease inhibitor (Thermo Fisher Scientific). Protein concentration of lysates was measured by the Bradford protein assay (BioRad) and equal amounts separated in 12% Tris-Glycine SDS-PAGE. Proteins were transferred to nitrocellulose membranes by semi-dry transfer. Membranes were blocked in TBST (Tris-buffered saline, 0.1% Tween 20) with 1% milk at room temperature for 1 hr. Primary antibodies (in 5% BSA/TBST) were incubated overnight at 4°C and detected the following day with secondary HRP conjugates (Jackson ImmunoResearch) using ECL (LumiGLO, Cell Signalling Technology) with film. The following primary antibodies were used for immunoblotting: Proteintech Group: MRPP3 (20959-1-AP, 1:3000); Abcam/Mitosciences: MT-CO1 (1D6E1A8, 1:500) and SDHA (C2061/ab14715, 1:10000); Santa Cruz: TOM40 (sc-11414, 1:5000) and Thermo Fisher Scientific: MRPP1 (A304-390A, 1:1000).

Immunohistochemistry

The NIH Animal Care and Use Committee approved protocol 1263-15 to T.B.F. for mice. C57/BJ6 mice at ages P04, P16 and P24 were euthanised, the cochleae were removed and fixed with 4% paraformaldehyde in PBS for 2 hours. The samples were microdissected and the organ of Corti was permeabilised with 0.5% Triton X-100 in PBS for 30 min followed by three 10 min washes with 1X PBS. Nonspecific binding sites were blocked with 5% normal goat serum and 2% BSA in PBS for 1 h at room temperature. Samples were incubated for 2 h

with rabbit polyclonal PRORP antibody (MRPP3, Proteintech, 20959-1-AP) at 1µg/ml and mouse monoclonal SNAP25 antibody (Santa Cruz, sc-136267) at 1µg/ml or rabbit polyclonal Tom20 antibody (Santa Cruz, sc-11415) at 2µg/ml and mouse monoclonal SNAP25 (Santa Cruz, sc-136267) at 1µg/ml followed by several rinses with PBS. Samples were incubated with goat anti-rabbit IgG Alexa Fluor 488 conjugated secondary antibody and goat anti mouse Alexa Fluor 568 conjugated secondary antibody (Molecular Probes) for 30 min. Samples were washed several times with PBS, with ProLongGold Antifade reagent with DAPI (Molecular Probes) and examined using an LSM780 confocal microscope (Zeiss Inc) equipped with 63X, 1.4 N.A. objective.

Acknowledgements

This study was supported by Action on Hearing Loss (S35); Action Medical Research (GN2494); NIHR Manchester Biomedical Research Centre ((IS-BRC-1215-20007); Wellcome Trust ISSF pump-prime award (097820/Z/11/B); the Wellcome Trust Centre for Mitochondrial Research (203105/Z/16/Z to RWT); the UK NHS Highly Specialised “Rare Mitochondrial Disorders of Adults and Children” Service (RWT); and The Lily Foundation (RWT); Austrian Science Fund (FWF) P25983 (WR); in part by the Intramural Research Program of the NIDCD at the NIH, (DC000039 to TBF); the Sigrid Juselius Foundation Senior Investigator Award (BJB; grants from the Hesperia Foundation; the Asociación Española contra las Leucodistrofias (ALE-ELA España), the PERIS program URD-Cat SLT002/16/00174); the Center for Biomedical Research on Rare Diseases (CIBERER) (ACCI19-759 to A.P); Fundació La Marató de TV3 (595/C/2020); Instituto de Salud Carlos III (FIS PI20/00758) (co-funded by European Regional Development Fund. ERDF, a way to build Europe); the Instituto de Salud Carlos III (Sara Borrell, CD19/00221 to E.V.), co-funded by European Social Fund; ESF investing in your future, and the Ministerio de Ciencia e Innovación y Universidades (Juan de la Cierva, FJCI-2016-28811 to E.V.). We also thank the CERCA Program/Generalitat de Catalunya for institutional support. This research was made possible through access to the data and findings generated by the 100,000 Genomes Project. The 100,000 Genomes Project is managed by Genomics England Limited (a wholly owned company of the Department of Health and Social Care). The 100,000 Genomes Project is funded by the National Institute for Health Research and NHS England. The Wellcome Trust, Cancer Research UK and the Medical Research Council have also funded research infrastructure. The 100,000 Genomes Project uses data provided by patients and collected by the National Health Service as part of their care and support. Thanks to Christie Boswell-Patterson for providing information from the Care4Rare Study. This work was performed under the Care4Rare Canada Consortium funded by Genome Canada and the Ontario Genomics Institute (OGI-147), the Canadian Institutes of Health Research, Ontario Research Fund, Genome Alberta, Genome British Columbia, Genome Quebec, and Children’s Hospital of Eastern Ontario Foundation.

References

1. Action on Hearing Loss. (2017). Levels of hearing loss. In. (Action on hearing loss.
2. Aiman, J., and Smentek, C. (1985). Premature ovarian failure. Obstet Gynecol 66, 9-14.
3. Reinhard, L., Sridhara, S., and Hallberg, B.M. (2015). Structure of the nuclease subunit of human mitochondrial RNase P. Nucleic Acids Res 43, 5664-5672.
4. Banka, S., Blom, H.J., Walter, J., Aziz, M., Urquhart, J., Clouthier, C.M., Rice, G.I., de Brouwer, A.P., Hilton, E., Vassallo, G., et al. (2011). Identification and characterization of an inborn error of metabolism caused by dihydrofolate reductase deficiency. Am J Hum Genet 88, 216-225.
5. Carr, I.M., Flintoff, K.J., Taylor, G.R., Markham, A.F., and Bonthron, D.T. (2006). Interactive visual analysis of SNP data for rapid autozygosity mapping in consanguineous families. Hum Mutat 27, 1041-1046.
6. Smith, M.J., Beetz, C., Williams, S.G., Bhaskar, S.S., O'Sullivan, J., Anderson, B., Daly, S.B., Urquhart, J.E., Bholah, Z., Oudit, D., et al. (2014). Germline mutations in SUFU cause Gorlin syndrome- associated childhood medulloblastoma and redefine the risk associated with PTCH1 mutations. J Clin Oncol 32, 4155-4161.
7. Sherry, S.T., Ward, M.H., Kholodov, M., Baker, J., Phan, L., Smigielski, E.M., and Sirotkin, K. (2001). dbSNP: the NCBI database of genetic variation. Nucleic Acids Res 29, 308-311.
8. NHLBI GO Exome Sequencing Project (ESP). Exome Variant Server. In. (Seattle, WA).
9. Turnbull, C., Scott, R.H., Thomas, E., Jones, L., Murugaesu, N., Pretty, F.B., Halai, D., Baple, E., Craig, C., Hamblin, A., et al. (2018). The 100 000 Genomes Project: bringing whole genome sequencing to the NHS. BMJ 361, k1687.
10. Kernohan, K.D, Hartley, T., Alirezaie, N., Care4Rare Canada Consortium, Robinson, P.N., Dyment, D.A., Boycott, K.M. (2018). Evaluation of exome filtering techniques for the analysis of clinically relevant genes. Hum Mutat 39:197-201.
11. Deutschmann, A.J., Amberger, A., Zavadil, C., Steinbeisser, H., Mayr, J.A., Feichtinger, R.G., Oerum, S., Yue, W.W., and Zschocke, J. (2014). Mutation or knock-down of 17beta-hydroxysteroid dehydrogenase type 10 cause loss of MRPP1 and impaired processing of mitochondrial heavy strand transcripts. Hum Mol Genet 23, 3618-3628.
12. Holzmann, J., Frank, P., Loffler, E., Bennett, K.L., Gerner, C., and Rossmanith, W. (2008). RNase P without RNA: identification and functional reconstitution of the human mitochondrial tRNA processing enzyme. Cell 135, 462-474.
13. Kunkel, T.A., Roberts, J.D., and Zakour, R.A. (1987). Rapid and efficient site-specific mutagenesis without phenotypic selection. Methods Enzymol 154, 367-382.
14. Rossmanith, W., Tullo, A., Potuschak, T., Karwan, R., and Sbisa, E. (1995). Human mitochondrial tRNA processing. J Biol Chem 270, 12885-12891.
