# Supplementary material for: Bi-allelic variants in the mitochondrial RNase P subunit PRORP cause mitochondrial tRNA processing defects and pleiotropic multisystem presentations
Source: Am J Hum Genet. 2021 Oct 28;108(11):2195–204. doi: 10.1016/j.ajhg.2021.10.002 (PMC8595931; doi:10.1016/j.ajhg.2021.10.002)
Supplement: Document S2. Article plus supplemental information [file mmc2.pdf]

Bi-allelic variants in the mitochondrial RNase P subunit PRORP cause mitochondrial tRNA processing defects and pleiotropic multisystem presentations

Irit Hochberg,[1,16,22] Leigh A.M. Demain,[2,3,22] Julie Richer,[4] Kyle Thompson,[5] Jill E. Urquhart,[2,3] Alessandro Rea,[2,3] Waheeda Pagarkar,[6] Agustí Rodríguez-Palmero,[7,8] Agatha Schlüter,[7] Edgard Verdura,[7] Aurora Pujol,[7,9] Pilar Quijada-Fraile,[10] Albert Amberger,[11] Andrea J. Deutschmann,[11] Sandra Demetz,[11] Meredith Gillespie,[4] Inna A. Belyantseva,[12] Hugh J. McMillan,[13] Melanie Barzik,[12] Glenda M. Beaman,[2,3] Reeya Motha,[14] Kah Ying Ng,[15] James O'Sullivan,[2,3] Simon G. Williams,[2,3] Sanjeev S. Bhaskar,[2,3] Isabella R. Lawrence,[5] Emma M. Jenkinson,[2] Jessica L. Zambonin,[4] Zeev Blumenfeld,[16] Sergey Yalonetsky,[13,17] Stephanie Oerum,[18] Walter Rossmanith,[19] Genomics England Research Consortium, Wyatt W. Yue,[18] Johannes Zschocke,[11] Kevin J. Munro,[20,21] Brendan J. Battersby,[15] Thomas B. Friedman,[12] Robert W. Taylor,[5] Raymond T. O'Keefe,[2,*] and William G. Newman[2,3,*]

Summary

Human mitochondrial RNase P (mt-RNase P) is responsible for 5′ end processing of mitochondrial precursor tRNAs, a vital step in mitochondrial RNA maturation, and is comprised of three protein subunits: TRMT10C, SDR5C1 (HSD10), and PRORP. Pathogenic variants in *TRMT10C* and *SDR5C1* are associated with distinct recessive or x-linked infantile onset disorders, resulting from defects in mitochondrial RNA processing. We report four unrelated families with multisystem disease associated with bi-allelic variants in *PRORP*, the metallonuclease subunit of mt-RNase P. Affected individuals presented with variable phenotypes comprising sensorineural hearing loss, primary ovarian insufficiency, developmental delay, and brain white matter changes. Fibroblasts from affected individuals in two families demonstrated decreased steady state levels of PRORP, an accumulation of unprocessed mitochondrial transcripts, and decreased steady state levels of mitochondrial-encoded proteins, which were rescued by introduction of the wild-type PRORP cDNA. In mt-tRNA processing assays performed with recombinant mt-RNase P proteins, the disease-associated variants resulted in diminished mitochondrial tRNA processing. Identification of disease-causing variants in *PRORP* indicates that pathogenic variants in all three subunits of mt-RNase P can cause mitochondrial dysfunction, each with distinct pleiotropic clinical presentations.

Mitochondrial RNase P (mt-RNase P) is the endonuclease that processes the 5′ end of mitochondrial tRNAs and thereby also releases adjacent mRNAs and rRNAs from the polycistronic primary transcripts.[1] In humans, the mt-RNase P complex is composed of three proteins, TRMT10C, SDR5C1 (HSD10), and PRORP (called MRPP1, MRPP2, and MRPP3, respectively), each encoded by the nuclear genome.[2,3] Bi-allelic variants in *TRMT10C* (MIM: 615423) have been identified in two unrelated individuals with a lethal childhood multisystem disorder, characterized by muscle hypotonia, sensorineural hearing loss (SNHL), metabolic acidosis, and multiple oxidative phosphorylation (OXPHOS) deficiencies (MIM: 616974).[4] SDR5C1 (also known as HSD10, HADH2, MRPP2, or ABAD [MIM: 300256]), encoded by the X chromosome gene *HSD17B10*, is a moonlighting protein with involvement in multiple biochemical pathways, including isoleucine metabolism.[3,5] Pathogenic variants in *HSD17B10*

[1]Institute of Endocrinology, Diabetes, and Metabolism, Rambam Health Care Campus, Haifa 3109601, Israel; [2]Division of Evolution, Infection, and Genomics, School of Biological Sciences, University of Manchester, Manchester M13 9PL, UK; [3]Manchester Centre for Genomic Medicine, St Mary's Hospital, Manchester University NHS Foundation Trust, Manchester M13 9WL, UK; [4]Department of Genetics, Children's Hospital of Eastern Ontario, Ottawa, ON K1H 8L1, Canada; [5]Wellcome Centre for Mitochondrial Research, Clinical and Translational Research Institute, Faculty of Medical Sciences, Newcastle University, Newcastle upon Tyne NE2 4HH, UK; [6]Royal National ENT and Eastman Dental Hospital, University College London Hospitals, London WC1E 6DG, UK; [7]Neurometabolic Diseases Laboratory, Bellvitge Biomedical Research Institute, L'Hospitalet de Llobregat, and Center for Biomedical Research on Rare Diseases, 08908 Barcelona, Spain; [8]Paediatric Neurology Unit, Hospital Universitari Germans Trias i Pujol, Universitat Autònoma de Barcelona, 08916 Barcelona, Spain; [9]Catalan Institution for Research and Advanced Studies, 08010 Barcelona, Spain; [10]Unit of Mitochondrial and Inherited Metabolic Diseases, Pediatric Department, University Hospital 12 de Octubre, National Reference Center, European Reference Network for Hereditary Metabolic Disorders, 28041 Madrid, Spain; [11]Institute of Human Genetics, Medical University Innsbruck, Innsbruck 6020, Austria; [12]Laboratory of Molecular Genetics, National Institute on Deafness and Other Communication Disorders, National Institutes of Health, Bethesda, MD 20892-3729, USA; [13]Department of Pediatrics, Children's Hospital of Eastern Ontario, University of Ottawa, Ottawa, ON K1H 8L1, Canada; [14]The Royal London Hospital, Whitechapel Road, Whitechapel, London E1 1FR, UK; [15]Institute of Biotechnology, University of Helsinki, 00790 Helsinki, Finland; [16]Rappaport Faculty of Medicine, Technion - Israel Institute of Technology, Haifa 3109601, Israel; [17]Department of Pediatric Cardiology, Rambam Health Care Campus, Haifa 3109601, Israel; [18]Newcastle MX Structural Biology Laboratory, Newcastle University, Medical School, NUBI Framlington Place, Newcastle upon Tyne NE2 4HH, UK; [19]Center for Anatomy and Cell Biology, Medical University of Vienna, 1090 Vienna, Austria; [20]Manchester Centre for Audiology and Deafness, School of Health Sciences, University of Manchester, Manchester M13 9PL, UK; [21]Manchester University NHS Foundation Trust, Manchester M13 9WL, UK
[22]These authors contributed equally
*Correspondence: rokeefe@manchester.ac.uk (R.T.O.), william.newman@manchester.ac.uk (W.G.N.)
https://doi.org/10.1016/j.ajhg.2021.10.002.
© 2021 The Authors. This is an open access article under the CC BY license (http://creativecommons.org/licenses/by/4.0/).

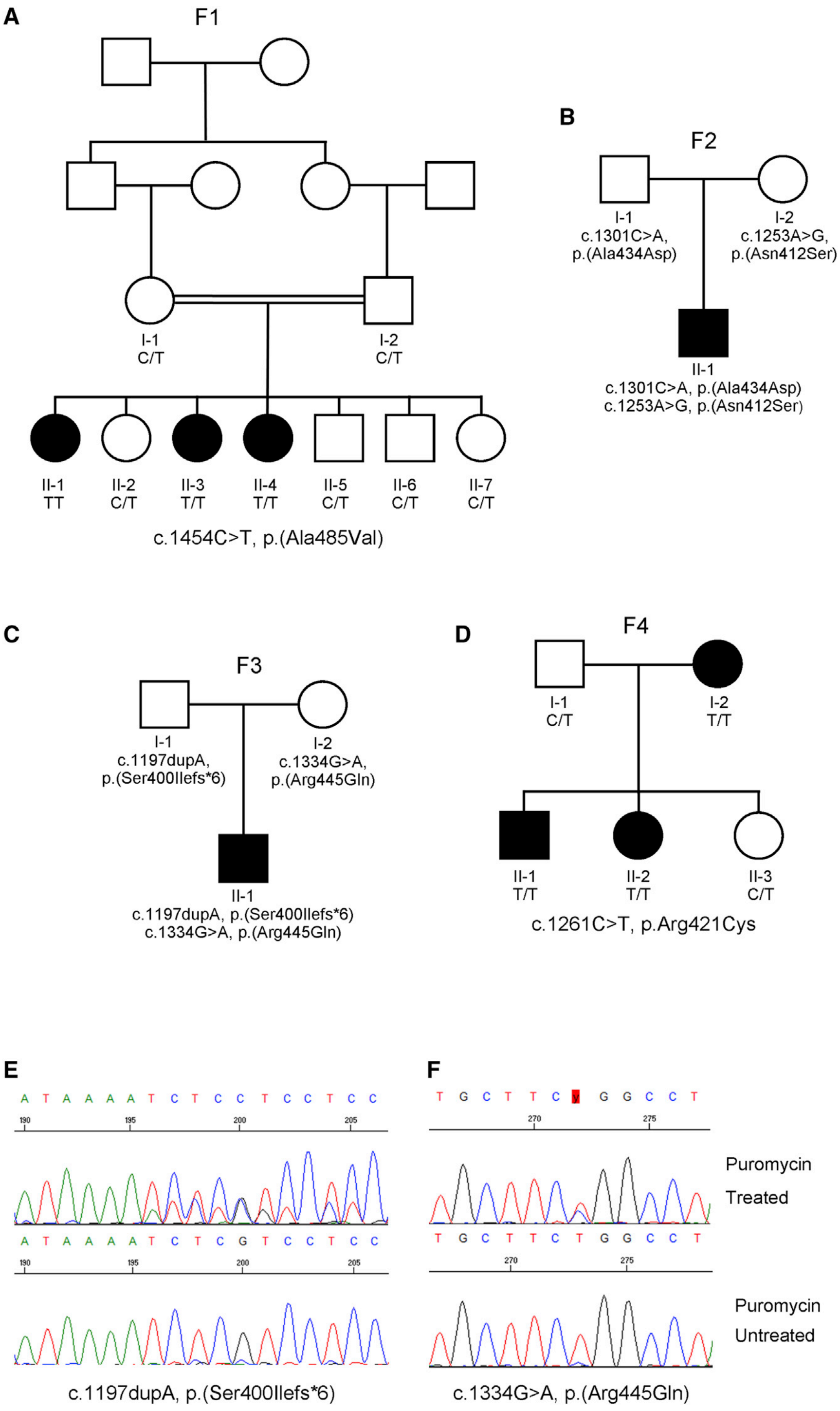

Figure 1. Variants in *PRORP* in four affected families result in pleiotropic clinical presentations

(A) The pedigree for a family (F1) with a variant in *PRORP*.

(B) The pedigree for the family F2.

(C) The pedigree for the family F3 and compound heterozygous variants in *PRORP*.

(D) The pedigree for a family (F4) with three affected individuals. Filled symbols indicate affected individuals.

(E and F) Sanger sequencing trace for the variants c.1197dupA and c.1334 G>A (highlighted in red) in the proband from F3 (II-1) with cDNA from puromycin treated and untreated fibroblasts. We display sequences in the reverse orientation to prevent masking of the missense variant by the frameshift variant. Sanger sequencing of the cDNA revealed that the frameshift c.1197dupA variant was present in the puromycin-treated cells, but not the untreated cells (E) and the missense variant c.1334G>A was present as a hemizygous change in the untreated samples (F), indicating that the frameshift transcript undergoes nonsense-mediated decay (E and F).

cause HSD10 disease (MIM: 300438), manifesting in males as a severe, infantile-onset neurodegenerative condition with cardiomyopathy.[5,6] Both disorders are characterized by defects of mitochondrial tRNA processing.[4,7,8]

PRORP (previously *KIAA0391* [MIM: 609947]) encodes the endonuclease subunit of the mt-RNase P complex. Here, we describe four families with overlapping phenotypes resulting from bi-allelic variants in *PRORP*. All individuals or their guardians provided written informed consent to participate in the gene discovery study in accordance with local regulations (see supplemental information).

Affected individuals from two families, F1 and F2, presented with SNHL, which was accompanied in the affected females in F1 by primary ovarian insufficiency, consistent with a diagnosis of Perrault syndrome (MIM: 233400).[9] In family F3, there was childhood onset of SNHL, lactic acidosis, and leukoencephalopathy, whereas affected individuals in family F4 presented with leukoencephalopathy. Recent reports of some individuals with variants in genes associated with Perrault syndrome have expanded the phenotypic spectrum to include presentations with childhood metabolic crises[9,10] and leukoencephalopathy.[9,11]

Family 1 (F1) is composed of three affected female siblings, two unaffected female siblings, two unaffected male siblings, and their unaffected parents (Figure 1A). At the last assessment the affected sisters were aged 30, 28, and 26 years of age. All three affected sisters presented with absent middle ear acoustic reflex, despite normal tympanometry, when tested in infancy and subsequent audiology examinations in each sister revealed profound bilateral SNHL (>90 dB hearing level at all frequencies) (Figure S1A). The three affected sisters each presented in their late teenage years with primary

amenorrhea, consistent with a diagnosis of Perrault syndrome (see “GeneReviews” in web resources). Pelvic ultrasound noted the absence of ovarian tissue in all three sisters. Hormonal profiles indicated hypergonadotropic hypogonadism (Figure S1C) with otherwise normal endocrine and biochemical tests and a 46, XX karyotype. The affected sisters were prescribed estrogen to induce puberty and are currently maintained on hormone replacement therapy. Each affected sibling has mild non-progressive intellectual disability (brain MR imaging has not been performed). Echocardiography for each of the affected sisters was normal. All other physical and neurological examinations were normal.

A homozygous variant in *PRORP*, c.1454C>T (p.Ala485Val) (GenBank: NM_014672.3), was identified in the affected individuals of F1 via autozygosity mapping and whole-exome sequencing. The variant segregated with the phenotype in the family.

Family 2 (F2) comprises a male proband and his unaffected, unrelated parents (Figure 1B). The proband (F2, II-1) was born at 40 + 4 weeks by emergency caesarean section for fetal tachycardia and meconium stained liqor. Hearing loss in the proband was first noted at 3 years of age but formally diagnosed at 5 years, at which age his brain magnetic resonance imaging (MRI) was normal. He was 9 years at last assessment and had bilateral mild to moderate SNHL (Figure S1B). His speech and language skills are delayed as a result of the hearing loss and he wears bilateral hearing aids. No behavioral or neurological issues have been noted and cardiovascular, respiratory, and abdominal system examinations have been unremarkable. A maternally inherited c.1235A>G (p.Asn412Ser) variant and a paternally inherited c.1301C>A (p.Ala434Asp) variant in *PRORP* were identified in the proband from whole genome sequence data generated through the 100,000 Genomes Project (Figure 1B).[12]

In family 3 (F3), a male proband (F3, II-1) of non-consanguineous unaffected parents (Figure 1C) was born by emergency caesarean section for failure to progress at 41 + 2 weeks gestation. Examination shortly after birth found appendicular hypertonia, more pronounced on the left-hand side, and mild dysmorphism (mild hypertelorism, bilateral epicanthal folds, thin vermilion of the lips, and microretrognathia). At 7 months of age, plasma lactic acid levels were increased at 5.6 mmol/L (reference 0.5–2.5 mmol/L) despite normal levels of plasma amino acids, urine organic acids, acylcarnitines, and free and total carnitine. Urine analysis and serum creatinine were normal. Repeat testing at 24 months revealed plasma lactic acid levels still raised at 3.0 mmol/L. Severe feeding difficulties resulted in the insertion of a gastrostomy tube at 15 months. An electroencephalogram (photic stimulation included) at 19 months revealed no evidence of seizure activity. At 20 months, he had severe global developmental delay, diffuse asymmetric hypertonia, acquired microcephaly (head circumference: 45.7 cm, $<2^{nd}$ percentile), and a mild scoliosis. Brain MRI at 13 months revealed periventricular nodular heterotopias, a dysplastic corpus callosum, diffuse sub-cortical white matter loss, and bilateral connatal cysts (Figure S2). Audiological tests were normal at 18 months, but at 3 years, an auditory brainstem response examination demonstrated evidence of auditory neuropathy spectrum disorder consistent with bilateral SNHL.

Microarray analysis performed on the proband (F3, II-1) detected no copy number variants. Whole-exome sequencing identified bi-allelic variants in *PRORP,* a maternally inherited missense variant c.1334G>A (p.Arg445Gln) (rs777185638) and a paternal frameshift variant c.1197dupA (p.Ser400Ilefs*6) (rs764714439), which was shown to result in nonsense-mediated decay of the transcript (Figures 1E and 1F).

Family F4 is a family comprising three affected individuals, including a brother and sister and their affected mother (Figure 1D). The proband (F4, II-1) was 19 years of age at last assessment. He presented with a psychotic disorder, autistic traits, and learning disability at 7 years. At 8 years, he presented with brief generalized seizures consisting of loss of consciousness and generalized stiffening of the body and extremities. The EEG was normal, but he received treatment with levetiracetam with a good response. Recent physical examination showed obesity and genu and talus valgus. Fundoscopy displayed papillary pallor.

Brain MRI indicated bilateral multiple periventricular and subcortical T2 white matter hyperintense lesions with a posterior predominance that remain unchanged in successive controls (Figure S2). Spectroscopy was normal. Electromyogram and nerve conduction studies revealed no evidence of neuromuscular abnormalities. Ocular and auditory nerve response as assessed by visual evoked potential and auditory brainstem response were normal. Metabolic studies indicated increased lactate/pyruvate ratio with normal plasma lactate, plasma amino acids, and urine organic acids. The proband is currently treated with coenzyme Q10, vitamin B2, vitamin C, carnitine, and arginine.

The affected sister of the proband (F4, II-2) was aged 17 years at last assessment. F4, II-2 presented with intrauterine growth retardation, global developmental delay, and seizures in the first years of life. At the age of 15 years, she presented with tremor in her legs, migraines, and hyperglycemia. Lactate levels were normal. Her hearing is normal as are her electromyogram (EMG) and nerve conduction studies. Brain MRI displayed bilateral multiple periventricular and subcortical T2 white matter hyperintense lesions with a posterior predominance that remain unchanged in successive controls (Figure S2). Spectroscopy was normal. Like her brother, she is treated with coenzyme Q10, vitamin B2, vitamin C, and carnitine.

The mother (F4, I-2) of the proband presented with retrobulbar optic neuritis and tonic pupil (a dilated pupil that responded slowly to light) at 39 years of age. Subsequently, she presented with asthenia, myalgias, memory

Table 1. Analysis of variants in *PRORP* in four families with distinct clinical presentations

Family	F1	F2		F3		F4
**Family details**						
Family details	three affected female siblings	one affected male		one affected male		two affected siblings and their affected mother
Phenotype	Perrault syndrome	SNHL		developmental delay, SNHL, lactic acidosis, and white matter changes		white matter changes
**Variant details**						
Variant	*PRORP*: c.1454C>T (p.Ala485Val)	*PRORP*: c.1235A>G (p.Asn412Ser)	*PRORP*: c.1301C>A (p.Ala434Asp)	*PRORP*: c.1334G>A (p.Arg445Gln)	*PRORP*: c.1197dupA (p.Ser400IlefsX6)	*PRORP*: c.1261C>T (p.Arg421Cys)
Location	Chr14(GRCh37): g.35739636C>T	Chr14(GRCh37): g.35649943A>G	Chr14(GRCh37): g.35735958C>A	Chr14(GRCh37): g.35735991G>A	Chr14(GRCh37): g.35649905dup	Chr14(GRCh37): g.35649969C>T
dbSNP	not present	rs148259590	rs144536804	rs777185638	rs764714439	rs147065101
Zygosity	homozygous	heterozygous	heterozygous	heterozygous	heterozygous	homozygous
Inheritance	N/A	maternal	paternal	maternal	paternal	N/A
gnomAD MAF (count)	not present	0.0001382 (39 heterozygotes, 0 homozygotes)	0.0008385 (237 heterozygotes, 0 homozygotes)	0.00002475 (7 heterozygotes, 0 homozygotes)	0.00003593 (9 heterozygotes, 0 homozygotes)	0.0001279 (36 heterozygotes, 0 homozygotes)
**Prediction tools**						
SIFT	deleterious (0.0)	deleterious (0.0)	deleterious (0.03)	deleterious (0.0)	N/A	deleterious (0.03)
PolyPhen	probably damaging (1.0)	probably damaging (1.0)	benign (0.443)	probably damaging (1.0)	N/A	probably damaging (1.0)
MutationTaster	disease causing (1.0)	disease causing (1.0)	disease causing (0.811)	disease causing (1.0)	N/A	disease causing (1.0)
VarCards	0.91 (extreme)	0.74(extreme)	0.39	0.83 (extreme)	N/A	0.57 (extreme)
CADD	34	26.4	28.2	34	N/A	35
Conservation	highly conserved	highly conserved	moderately conserved	highly conserved	N/A	highly conserved

(*Continued on next page*)

Table 1. Continued

Family	F1	F2	F3	F4
**Effect on protein**				
Variant prediction	may distort the active site	may interfere with the shape of the active site	reduce the stability of the protein	directly impact nuclease function	loss of function	this region is disordered in the structure so was not modeled
FOLDX change	reduces stability ($\Delta\Delta G$ 3.97 kcal/mol)	slightly reduces stability ($\Delta\Delta G$ 1.61 kcal/mol)	slightly reduces stability ($\Delta\Delta G$ 1.66 kcal/mol)	slightly reduces stability ($\Delta\Delta G$ 2.32 kcal/mol)	N/A	N/A
tRNA processing (approximate % of wild-type)	82%	13%	86%	25%	N/A	81%

All variants mapped to the *PRORP* transcript GenBank: NM_014672.3. SNHL, sensorineural hearing loss; MAF, minor allele frequency.

loss, and frequent headaches. She also had two episodes of left hemiparesis and hypoesthesia that resolved in 15 days without specific treatment and repetitive episodes of lower limb thrombophlebitis. Examination revealed afferent pupillary defect, nasal hemianopsia of the right visual field, and abnormal color perception. She also had distal weakness of the lower limbs, areflexia, and vibratory hypoesthesia of the left side of her body. Her hearing is normal, and she has no evidence of ovarian insufficiency. Routine blood tests and metabolic and thrombophilia studies were all normal. Specific genetic testing for metachromatic leukodystrophy, Krabbe disease, CADASIL, and Leber optic neuropathy were negative. Cerebrospinal fluid (CSF) oligoclonal bands and anti-MOG and anti-NMO antibodies were also negative. Visual evoked potentials displayed abnormal conduction in the right eye. EMG and nerve conduction studies were normal. Brain MRI indicated bilateral multiple periventricular and subcortical T2 white matter hyperintense lesions affecting both hemispheres, corpus callosum, pons, and cerebellum with no contrast enhancement and no changes in successive controls (Figure S2).

Whole-genome sequencing identified a homozygous c.1261C>T (p.Arg421Cys) *PRORP* variant in all three affected individuals. The father (F4, I-1) of the two affected children was a carrier for the variant, and subsequently, the family was confirmed to be consanguineous, consistent with the pseudo-dominant inheritance pattern. There was no evidence of any other putative disease-associated variants in the genome and exome datasets in the four families when the filtering steps were applied (supplemental information).

The altered residues in PRORP identified in the affected individuals from the four families are all highly conserved from vertebrates to fly (Figure S3). All of the variants are either absent from gnomAD[13] or have a very low minor allele frequency and are not present as homozygous variants. All missense variants were predicted to be deleterious by multiple prediction software (Table 1). Of note, homozygous loss-of-function variants in *PRORP* are absent from publicly available databases and from a consanguineous cohort of >3,200 British Pakistani individuals.[14]

Mitochondrial tRNAs (mt-tRNAs) are processed at the 5′ end by mt-RNase P[1] and at the 3′ end by mt-RNase Z (encoded by *ELAC2* [MIM: 605367]).[15,16] This tRNA cleavage also releases most of the RNA species from the polycistronic mitochondrial precursor transcripts according to the mitochondrial tRNA punctuation model.[17,18] PRORP, as a subunit of mt-RNase P, catalyzes the Mg^{2+}-dependent phosphodiester-bond cleavage of 5′ extensions of mitochondrial tRNAs.[2,19] The processing of mitochondrial tRNAs proceeds in a stepwise manner and 5′ cleavage by mt-RNase P precedes tRNA 3′ end processing.[1,18]

We investigated the steady-state levels of the mt-RNase P subunits TRMT10C, SDR5C1, and PRORP in dermal fibroblasts available from affected individuals in families F1 and F3 by immunoblotting and detected a decrease in

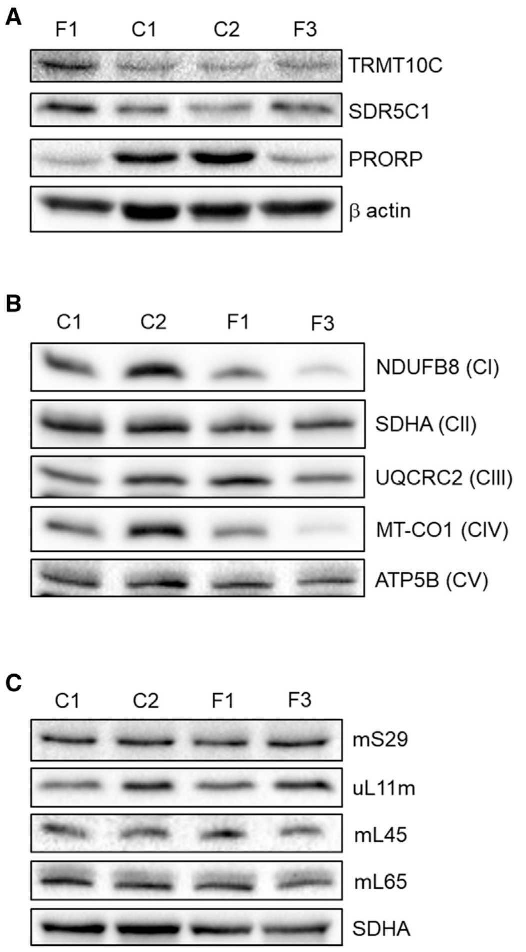

Figure 2. Fibroblasts from affected individuals F1, II-4 and F3, II-1 display reduction in subunits of mt-RNase P and reduced levels of mitochondrial DNA encoded-OXPHOS subunits but no reduction in mitochondrial ribosomal proteins
(A) Immunoblot analysis of mt-RNase P subunits TRMT10C, SDR5C1, and PRORP in fibroblasts from two healthy controls (C1 and C2); individual F1, II-4 with the p.Ala485Val variant in PRORP and the individual F3, II-2, who has compound heterozygous variants in *PRORP* (n = 3)
(B) Immunoblot analysis of proteins of the five oxidative phosphorylation complexes. Included are two control samples (C1 and C2) and two samples from affected individuals as detailed in (A) (n = 3).
(C) Immunoblot analysis of protein subunits of the mitochondrial ribosome. SDHA is included as a loading control. Samples for affected individuals and controls labeled as in (A) (n = 3).

PRORP levels in both affected individuals compared to controls (Figure 2A). The decrease in PRORP suggests that the variant p.Ala485Val is either less stable than the wild-type protein or downregulated in affected individuals from family F1. The decreased PRORP levels in F3, II-1 may partially result from absence of protein due to the allele that is subject to nonsense-mediated decay (Figures 1E and 1F). We also detected decreased steady-state levels of respiratory chain complex I (NDUFB8) and complex IV (COXI) subunits in fibroblasts from affected individuals compared to controls—both complexes contain mitochondrial DNA-encoded subunits. There was no change of other OXPHOS components, most notably complex II, which is entirely nuclear encoded (Figure 2B). This profile is consistent with a generalized defect in mitochondrial translation. The decreased stability of complex I and IV subunits was more severe in subject F3, II-1, consistent with his more severe clinical phenotype. There was no noticeable difference in levels of several mitoribosomal proteins (MRPs) between the fibroblasts from affected individuals and controls (Figure 2C), indicating that any effect on translation most likely reflects a defect of transcript processing rather than a defect in the stability or assembly of the mitoribosome itself.

To determine the status of mitochondrial-encoded RNA transcripts in subject dermal fibroblasts, Northern blots were performed. We designed biotinylated strand-specific probes to detect transcripts from four different regions of the mitochondrial genome. An *MT-ND1* probe revealed the accumulation of a precursor RNA of approximately 2.5 kb in the samples from F1, II-4 and F3, II-1 (C1 and C2) (Figure 3), corresponding to unprocessed 16S rRNA-tRNALeu(UUR)-ND1 mRNA and apparently resulting from impaired 5′ end processing of mt-tRNA$^{Leu(UUR)}$. This RNA species was previously termed RNA 19 and observed to be upregulated by the 3243A>G MELAS and other variants in mt-tRNA$^{Leu(UUR)}$.[20–22] A larger RNA species was detected on a longer exposure for the *MT-ND1* probe, indicating that mt-tRNA^{Val} processing is also decreased. The *MT-ND2* and *MT-CO2* probes both detected multiple RNA species seen in affected individuals but not control samples. A *MT-ND6* probe detected a mitochondrial light strand transcript of approximately 2.3 kb in the F1, II-4 and F3, II-1 samples, which can be explained by impaired 5′ processing of mt-tRNA^{Glu} (Figure 3). The presence of multiple large transcripts in the samples from the affected individuals, not seen in the control samples at the same intensity, indicates a deficiency in 5′ processing across multiple mt-tRNA sites.

All disease-associated variants are located in the metallonuclease domain of PRORP (Figure S4A), as revealed by its crystal structure.[23] Residue Ala485 is situated close to four conserved aspartate residues implicated in metal-ion binding (Figure S4B).[23] Replacing the conserved alanine at residue 485 with the bulkier valine (F1) could distort the active site and impair catalysis by interfering with proper coordination of the metal ions, thereby reducing the endonucleolytic activity of PRORP. Residue Ala434 is surface exposed and the substitution Ala434Asp (F2) is predicted to slightly reduce the stability of PRORP but no structural change to the protein (Figure S4C). Residue Asn412 is located in the active site next to catalytic residue Asp409 (Figure S4D). Substitution Asn412Ser (also F2) results in no drastic structural changes but may interfere with the shape of the active site, thereby reducing the endonucleolytic activity of PRORP. Residue Arg445 forms stabilizing interactions with essential catalytic residues (e.g., Asp479, Asp478) (Figure S4E). It is likely that Arg445Gln (F3) would directly impact nuclease activity. In 150

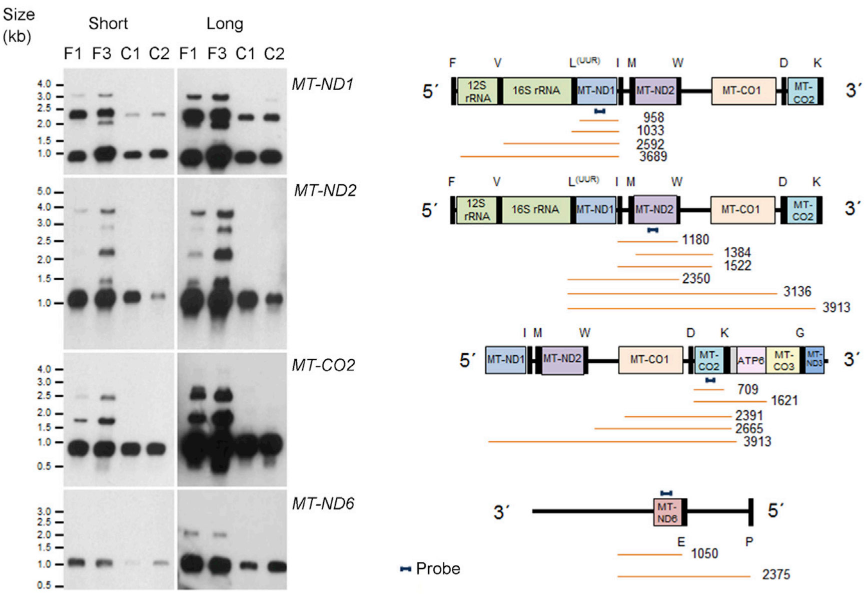

Figure 3. Fibroblasts from affected individuals display impaired mitochondrial RNA processing

(A) Northern blot assessment of RNA extracted from F1, II-4 and F3, II-1 fibroblasts and two control (C1 and C2) samples with strand specific probes designed to complement four different mitochondrial gene transcripts: *MT-ND1*, *MT-ND2*, *MT-CO2*, and *MT-ND6*. A long and short exposure of the blots are presented.

(B) Schematic representations of mitochondrial genome regions, the probes (red) and expected fragment sizes in bp (orange) are displayed to the right of each blot.

orthologs, the residue equivalent to Arg445 is invariably Arg (data not shown). The amino acid at residue 421 is highly variable and is disordered in the structure. During the review of this manuscript, the cryo-EM structure of PRORP in complex with tRNA, TRMT10C, and SDR5C1 was determined.[24] In addition to supporting the above interpretations of disease-associated variants, this structure reveals that PRORP residue Arg445 forms an interaction with the 5′ end of the tRNA substrate, which will be broken by Arg445Gln (F3). Additionally, Arg421 becomes ordered in the context of the complex and forms stabilizing interactions with residue Glu429, which will be disrupted by Arg421Cys (F4). We investigated whether the disease-associated variants in *PRORP* affected the catalytic activity of the mt-RNase P complex. The three mt-RNase P complex wild-type proteins (TRMT10C, SDR5C1, and PRORP), and the PRORP variant proteins, were individually produced by recombinant expression in bacteria and purified. Recombinant mt-RNase P was reconstituted *in vitro* and 5′ leader processing monitored with fluorescent labeled mt-pre-tRNA^{Ile}. All the amino acid variants in PRORP led to a reduction in mt-tRNA^{Ile} cleavage product compared to wild-type PRORP (Figure 4A). The fluorescence intensity of the mt-tRNA^{Ile} cleavage product was quantified. After 30 min from the start of the reaction, the mt-RNase P complexes with variants PRORP p.Arg445Gln (F3) and p.Asn412Ser (F2) displayed the most dramatic decreases in mt-tRNA^{Ile} cleavage products compared to wild-type of approximately 76% and 87%, respectively. The mt-RNase P complexes with variants PRORP p.Ala485Val (F1), p.Ala434Asp (F2), and p.Arg421Cys (F4) reduced 5′ leader processing by approximately 19%, 15%, and 10%, respectively. The reductions in processing persisted after 60 min (Table 1). These data indicate that disease-associated PRORP variants reduce the RNase P activity of the complex *in vitro*. Of note, the greatest reduction of activity is seen in the most severely clinically affected individual (F3, II-1), where a frameshift resulting in loss of function is present in combination with PRORP p.Arg445Gln. However, there are insufficient data to define genotype-phenotype correlations because of the small number of affected individuals ascertained and investigated.

We performed rescue experiments to establish whether expression of wild-type *PRORP* could reduce the accumulation of unprocessed transcripts in the fibroblasts from an affected individual. Fibroblasts from individual F3, II-1 were transduced with a retroviral vector containing the wild-type *PRORP* cDNA or wild-type *TRMT10C* cDNA as a control. Expression of wild-type *PRORP* restored both the amount of PRORP and MT-CO1 protein, whereas TRMT10C and SDHA levels were unaffected (Figure 4B). This result indicates that increased levels of wild-type PRORP in the cells from the affected individual enhances the steady state levels of mitochondrial-encoded MT-CO1 but does not affect levels of nuclear-encoded SDHA. This effect is not seen with the empty vector or *TRMT10C* (Figure 4C). Transducing fibroblasts from the affected individual with *PRORP* also decreased the levels of unprocessed mitochondrial transcripts in the cells to near wild-type levels (Figure 4B). Again, this effect is not seen with the empty vector or the vector containing the coding sequence for *TRMT10C*. Taken together, these data indicate that the expression of wild-type *PRORP* in cells from an affected individual can rescue the molecular defects.

We undertook localization studies of PRORP in the mouse organ of Corti to understand why variants in *PRORP* may be associated with hearing loss (Figure S5). After the onset of hearing, which occurs in mice at postnatal day 12, PRORP is detected around the afferent and efferent synapses of the inner hair cells and the efferent synapses of the outer hair cells, indicating possible importance for synaptic functions after the onset of hearing.

PRORP partially co-localizes with a synaptic marker (SNAP25), indicating that it may not only be present in the mitochondria of efferent synapses but possibly also

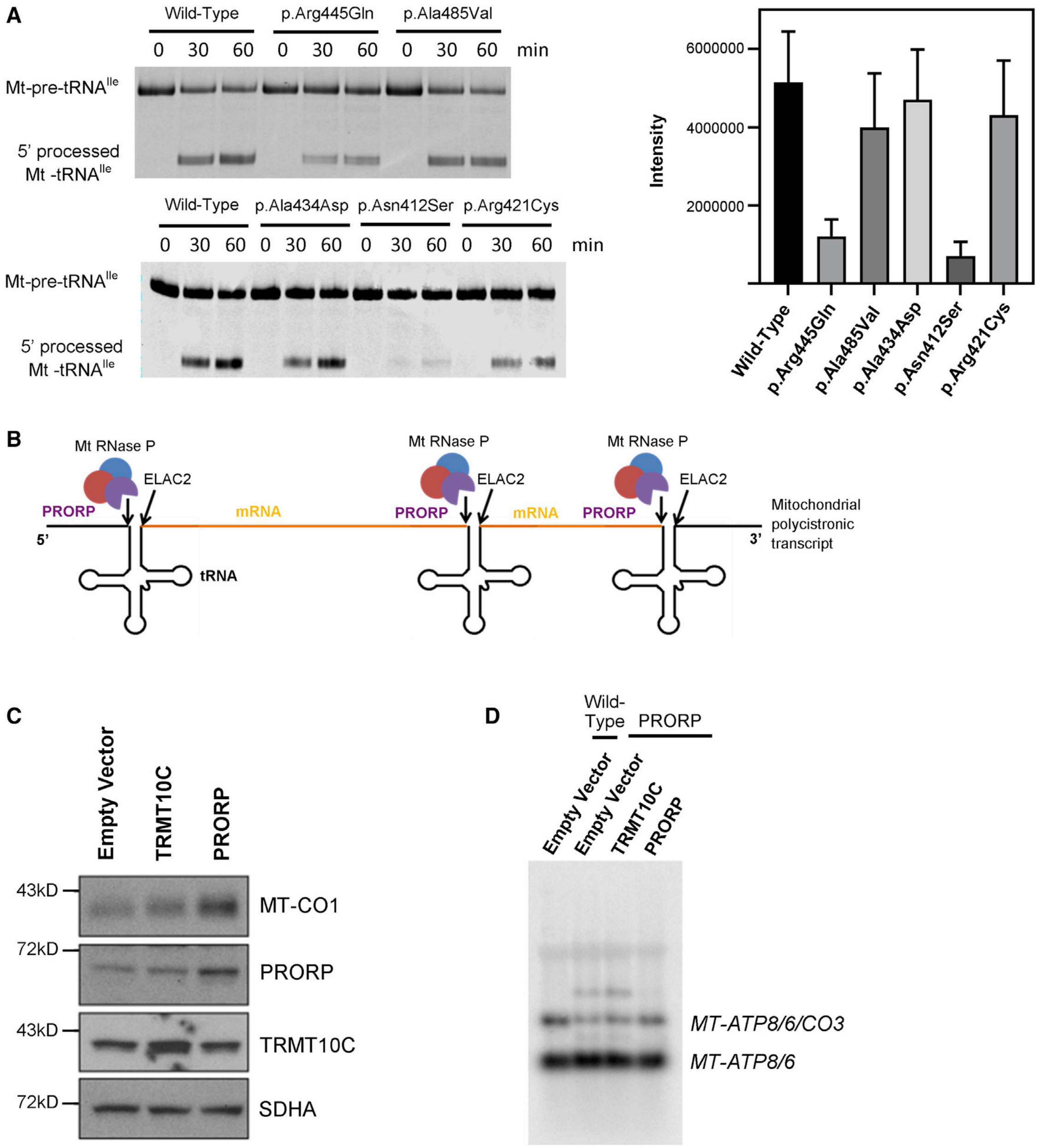

Figure 4. *In vitro* mt-RNase P processing assays reveal all variants produce less 5′-end-processed tRNA than wild-type PRORP; processing defects in subject F3 II-1 are rescued by wild-type *KIAA0391*

(A) Mitochondrial pre-tRNA^{Ile} was cleaved by reconstituted recombinant mt-RNase P containing either wild-type or variant *PRORP*, as indicated, resulting in the release of the 5′ leader sequence. Aliquots were taken from the reactions at the time points indicated and resolved by denaturing electrophoresis. Quantitative analysis of pre-tRNA^{Ile} processing revealed an overall decrease in fluorescence intensity of the processed tRNA between wild-type and variants over three replicate experiments. Error bars indicate standard deviation.
(B) Cartoon to illustrate the role of PRORP within the RNase P complex in 5′ end cleavage of mitochondrial tRNA transcripts.
(C) Immunoblotting of whole cell lysates from fibroblasts stably transduced with the indicated cDNAs. (C) Northern blotting of total RNA hybridized with a strand-specific oligonucleotide probe against *MT-ATP8*.

mitochondria of afferent synapses and nerve fibers around the inner hair cells. The pattern of PRORP staining does not entirely co-localize with mitochondrial marker TOM20. This lack of complete co-localization with TOM20 suggests that the high levels of PRORP found in a subset of mitochondria associated with the synapses and neurons of the organ of Corti hair cells reflect the increased demand for mitochondrial tRNA processing and translation in these cells, which may be a characteristic of a particular type of mitochondria at these locations.[25–27]

In summary, we present genetic and functional evidence that bi-allelic variants in *PRORP* are associated with pleiotropic clinical presentations and that *PRORP* should be considered another gene associated with the Perrault syndrome clinical spectrum. Such variability in clinical

presentation is not uncommon for mitochondrial disorders and is increasingly being shown for genes associated with Perrault syndrome.[9] Bi-allelic hypomorphic variants in *CLPP*, for example, are associated with Perrault syndrome,[28] whereas more deleterious variants result in a more severe phenotype associated with SNHL, seizures, and brain white matter changes.[11] The clinical spectrum observed in individuals with different bi-allelic *PRORP* variants is consistent with this phenotypic range and most likely reflects altered mitochondrial dysfunction in different tissues at different time points. It is important to note that *PRORP* is ubiquitously expressed in the GTEx dataset. This is consistent with many disorders of mitochondrial function, which have specific clinical phenotypes despite these expression profiles. Notably in the families with multiple affected individuals (F1 and F4), the phenotypes were consistent, indicating that certain *PRORP* variants may result in specific phenotypes.

Similar OXPHOS defects to those seen in individuals with *PRORP* variants have been observed in individuals with pathogenic variants in the mt-RNase P genes *TRMT10C*[4] and *HSD17B10*,[29] suggesting a common pathogenic mechanism in these disorders. Despite the similarities in defective mitochondrial tRNA processing, variants in the three subunits of mt-RNase P result in different clinical phenotypes. With our work, we demonstrate that bi-allelic variants in *PRORP* result in mitochondrial dysfunction and that all three subunits of mitochondrial RNase P have now been associated with mitochondrial disease, each with distinct pleiotropic clinical presentations.

Data and code availability

The *PRORP* variants were submitted to ClinVar (https://www.ncbi.nlm.nih.gov/clinvar/) (GenBank: NM_014672.4; accession numbers SCV001943322–SCV001943327). The exome and genome datasets supporting this study have not been deposited in a public repository because of ethical restriction but are available from the corresponding author on request.

Supplemental information

Supplemental information can be found online at https://doi.org/10.1016/j.ajhg.2021.10.002.

Acknowledgments

We would like to thank the families for their participation. Family F2 was ascertained via the 100,000 Genomes Project.[12] Families F3 and F4 were identified via GeneMatcher.[30] Further funding details are available in the supplemental information.

Declaration of interests

The authors declare no competing interests.

Received: August 24, 2021
Accepted: October 7, 2021
Published: October 28, 2021

Web resources

dbSNP, https://www.ncbi.nlm.nih.gov/projects/SNP/
Exome Variant Server, https://evs.gs.washington.edu/EVS/
FoldX, http://foldxsuite.crg.eu/
GenBank, https://www.ncbi.nlm.nih.gov/genbank/
GeneMatcher, https://genematcher.org/
GeneReviews, Newman, W.G., Friedman, T.B., Conway, G.S., and Demain, L.A.M. (2018). Perrault Syndrome, https://www.ncbi.nlm.nih.gov/books/NBK242617/
gnomAD, https://gnomad.broadinstitute.org/
GTEx, https://gtexportal.org/home/
LOVD, https://www.lovd.nl/
MutationTaster, http://www.mutationtaster.org/
OMIM, https://www.omim.org/
PolyPhen-2, http://genetics.bwh.harvard.edu/pph2/
SIFT, https://sift.bii.a-star.edu.sg/

References

1. Rackham, O., Busch, J.D., Matic, S., Siira, S.J., Kuznetsova, I., Atanassov, I., et al. (2016). Hierarchical RNA processing is required for mitochondrial ribosome assembly. Cell Rep. *16*, 1874–1890.
2. Holzmann, J., Frank, P., Löffler, E., Bennett, K.L., Gerner, C., and Rossmanith, W. (2008). RNase P without RNA: identification and functional reconstitution of the human mitochondrial tRNA processing enzyme. Cell *135*, 462–474.
3. Vilardo, E., Nachbagauer, C., Buzet, A., Taschner, A., Holzmann, J., and Rossmanith, W. (2012). A subcomplex of human mitochondrial RNase P is a bifunctional methyltransferase–extensive moonlighting in mitochondrial tRNA biogenesis. Nucleic Acids Res. *40*, 11583–11593.
4. Metodiev, M.D., Thompson, K., Alston, C.L., Morris, A.A.M., He, L., Assouline, Z., et al. (2016). Recessive mutations in TRMT10C cause defects in mitochondrial RNA processing and multiple respiratory chain deficiencies. Am. J. Hum. Genet. *98*, 993–1000.
5. Zschocke, J. (2012). HSD10 disease: clinical consequences of mutations in the HSD17B10 gene. J. Inherit. Metab. Dis. *35*, 81–89.
6. Ofman, R., Ruiter, J.P., Feenstra, M., Duran, M., Poll-The, B.T., Zschocke, J., Ensenauer, R., Lehnert, W., Sass, J.O., Sperl, W., and Wanders, R.J. (2003). 2-Methyl-3-hydroxybutyryl-CoA dehydrogenase deficiency is caused by mutations in the HADH2 gene. Am. J. Hum. Genet. *72*, 1300–1307.
7. Deutschmann, A.J., Amberger, A., Zavadil, C., Steinbeisser, H., Mayr, J.A., Feichtinger, R.G., Oerum, S., Yue, W.W., and Zschocke, J. (2014). Mutation or knock-down of 17β-hydroxysteroid dehydrogenase type 10 cause loss of MRPP1 and impaired processing of mitochondrial heavy strand transcripts. Hum. Mol. Genet. *23*, 3618–3628.
8. Vilardo, E., and Rossmanith, W. (2015). Molecular insights into HSD10 disease: impact of SDR5C1 mutations on the human mitochondrial RNase P complex. Nucleic Acids Res. *43*, 5112–5119.
9. Faridi, R., Rea, A., Fenollar-Ferrer, C., O'Keefe, R.T., Gu, S., Munir, Z., Khan, A.A., Riazuddin, S., Hoa, M., Naz, S., et al. (2021). New insights into Perrault syndrome, a clinically and genetically heterogeneous disorder. Hum. Genet. epub. https://doi.org/10.1007/s00439-021-02319-7.
10. Riley, L.G., Rudinger-Thirion, J., Frugier, M., Wilson, M., Luig, M., Alahakoon, T.I., Nixon, C.Y., Kirk, E.P., Roscioli, T., Lunke,

S., et al. (2020). The expanding LARS2 phenotypic spectrum: HLASA, Perrault syndrome with leukodystrophy, and mitochondrial myopathy. Hum. Mutat. *41*, 1425–1434.
11. Theunissen, T.E., Szklarczyk, R., Gerards, M., Hellebrekers, D.M., Mulder-Den Hartog, E.N., Vanoevelen, J., et al. (2016). Specific MRI abnormalities reveal severe Perrault syndrome due to CLPP defects. Front. Neurol. *7*, 203.
12. Turnbull, C., Scott, R.H., Thomas, E., Jones, L., Murugaesu, N., Pretty, F.B., Halai, D., Baple, E., Craig, C., Hamblin, A., et al. (2018). The 100 000 Genomes Project: bringing whole genome sequencing to the NHS. BMJ *361*, k1687.
13. Karczewski, K.J., Francioli, L.C., Tiao, G., Cummings, B.B., Alföldi, J., Wang, Q., Collins, R.L., Laricchia, K.M., Ganna, A., Birnbaum, D.P., et al. (2020). The mutational constraint spectrum quantified from variation in 141,456 humans. Nature *581*, 434–443.
14. Narasimhan, V.M., Hunt, K.A., Mason, D., Baker, C.L., Karczewski, K.J., Barnes, M.R., Barnett, A.H., Bates, C., Bellary, S., Bockett, N.A., et al. (2016). Health and population effects of rare gene knockouts in adult humans with related parents. Science *352*, 474–477.
15. Brzezniak, L.K., Bijata, M., Szczesny, R.J., and Stepien, P.P. (2011). Involvement of human ELAC2 gene product in 3¢ end processing of mitochondrial tRNAs. RNA Biol. *8*, 616–626.
16. Rossmanith, W. (2011). Localization of human RNase Z isoforms: dual nuclear/mitochondrial targeting of the ELAC2 gene product by alternative translation initiation. PLoS ONE *6*, e19152.
17. Ojala, D., Montoya, J., and Attardi, G. (1981). tRNA punctuation model of RNA processing in human mitochondria. Nature *290*, 470–474.
18. Rossmanith, W. (2012). Of P and Z: mitochondrial tRNA processing enzymes. Biochim. Biophys. Acta *1819*, 1017–1026.
19. Reinhard, L., Sridhara, S., and Hällberg, B.M. (2015). Structure of the nuclease subunit of human mitochondrial RNase P. Nucleic Acids Res. *43*, 5664–5672.
20. Schon, E.A., Koga, Y., Davidson, M., Moraes, C.T., and King, M.P. (1992). The mitochondrial tRNA(Leu)(UUR)) mutation in MELAS: a model for pathogenesis. Biochim. Biophys. Acta *1101*, 206–209.
21. Bindoff, L.A., Howell, N., Poulton, J., McCullough, D.A., Morten, K.J., Lightowlers, R.N., Turnbull, D.M., and Weber, K. (1993). Abnormal RNA processing associated with a novel tRNA mutation in mitochondrial DNA. A potential disease mechanism. J. Biol. Chem. *268*, 19559–19564.
22. Koga, A., Koga, Y., Akita, Y., Fukiyama, R., Ueki, I., Yatsuga, S., and Matsuishi, T. (2003). Increased mitochondrial processing intermediates associated with three tRNA(Leu(UUR)) gene mutations. Neuromuscul. Disord. *13*, 259–262.
23. Howard, M.J., Lim, W.H., Fierke, C.A., and Koutmos, M. (2012). Mitochondrial ribonuclease P structure provides insight into the evolution of catalytic strategies for precursor-tRNA 5′ processing. Proc. Natl. Acad. Sci. USA *109*, 16149–16154.
24. Bhatta, A., Dienemann, C., Cramer, P., and Hillen, H.S. (2021). Structural basis of RNA processing by human mitochondrial RNase P. Nat. Struct. Mol. Biol. *28*, 713–723.
25. Delgado, T., Petralia, R.S., Freeman, D.W., Sedlacek, M., Wang, Y.X., Brenowitz, S.D., Sheu, S.H., Gu, J.W., Kapogiannis, D., Mattson, M.P., and Yao, P.J. (2019). Comparing 3D ultrastructure of presynaptic and postsynaptic mitochondria. Biol. Open *8*, bio044834.
26. Wong, H.C., Zhang, Q., Beirl, A.J., Petralia, R.S., Wang, Y.X., and Kindt, K. (2019). Synaptic mitochondria regulate haircell synapse size and function. eLife *8*, e48914.
27. Freeman, D.W., Petralia, R.S., Wang, Y.X., Mattson, M.P., and Yao, P.J. (2017). Mitochondria in hippocampal presynaptic and postsynaptic compartments differ in size as well as intensity. Matters (Zur) *2017*. https://doi.org/10.19185/matters.201711000009.
28. Jenkinson, E.M., Rehman, A.U., Walsh, T., Clayton-Smith, J., Lee, K., Morell, R.J., Drummond, M.C., Khan, S.N., Naeem, M.A., Rauf, B., et al. (2013). Perrault syndrome is caused by recessive mutations in CLPP, encoding a mitochondrial ATP-dependent chambered protease. Am. J. Hum. Genet. *92*, 605–613.
29. Chatfield, K.C., Coughlin, C.R., 2nd, Friederich, M.W., Gallagher, R.C., Hesselberth, J.R., Lovell, M.A., Ofman, R., Swanson, M.A., Thomas, J.A., Wanders, R.J.A., et al. (2015). Mitochondrial energy failure in HSD10 disease is due to defective mtDNA transcript processing. Mitochondrion *21*, 1–10.
30. Sobreira, N., Schiettecatte, F., Valle, D., and Hamosh, A. (2015). GeneMatcher: a matching tool for connecting investigators with an interest in the same gene. Hum. Mutat. *36*, 928–930.

The American Journal of Human Genetics, Volume *108*

Supplemental information

Bi-allelic variants in the mitochondrial RNase P subunit PRORP cause mitochondrial tRNA processing defects and pleiotropic multisystem presentations

Irit Hochberg, Leigh A.M. Demain, Julie Richer, Kyle Thompson, Jill E. Urquhart, Alessandro Rea, Waheeda Pagarkar, Agustí Rodríguez-Palmero, Agatha Schlüter, Edgard Verdura, Aurora Pujol, Pilar Quijada-Fraile, Albert Amberger, Andrea J. Deutschmann, Sandra Demetz, Meredith Gillespie, Inna A. Belyantseva, Hugh J. McMillan, Melanie Barzik, Glenda M. Beaman, Reeya Motha, Kah Ying Ng, James O'Sullivan, Simon G. Williams, Sanjeev S. Bhaskar, Isabella R. Lawrence, Emma M. Jenkinson, Jessica L. Zambonin, Zeev Blumenfeld, Sergey Yalonetsky, Stephanie Oerum, Walter Rossmanith, Genomics England Research Consortium, Wyatt W. Yue, Johannes Zschocke, Kevin J. Munro, Brendan J. Battersby, Thomas B. Friedman, Robert W. Taylor, Raymond T. O'Keefe, and William G. Newman

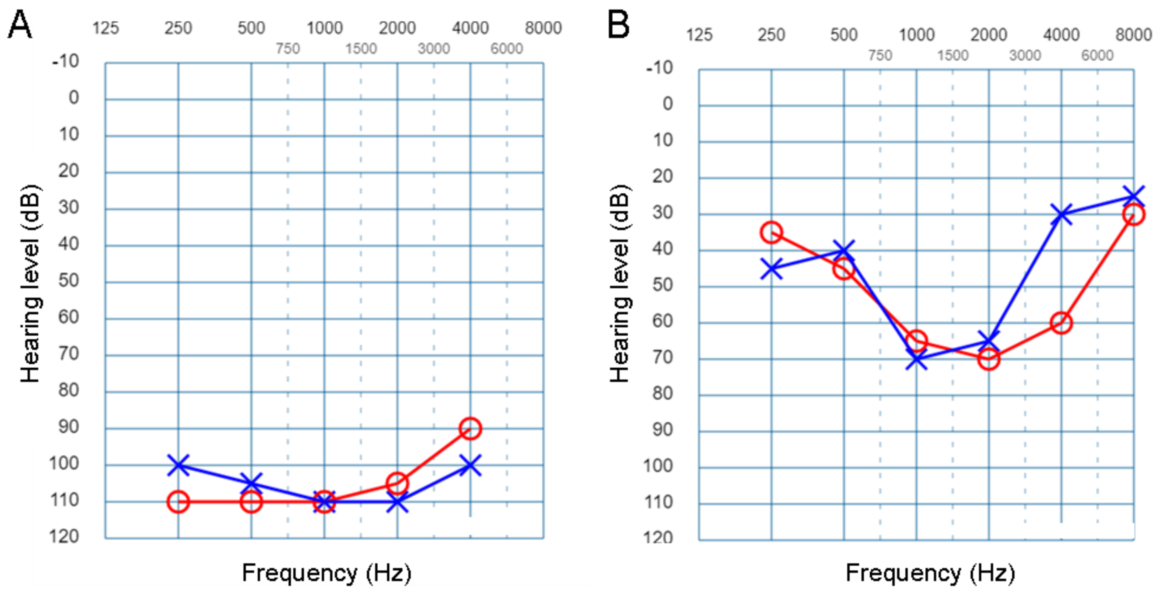

C

	II-1	II-3	II-4	Postmenopausal Reference Values
Follicle Stimulating Hormone (IU/l)	**90.7**	**53.5**	17.7	26.7 - 133.4
Luteinising Hormone (IU/l)	**31.8**	97.9	75.0	10.4 - 64.6
Estradiol (pmol/l)	14.2	**37.3**	12.0	36.7 - 102

Figure S1 – Affected individuals from family F1 and F2 have sensorineural hearing loss, and affected individuals from family F1 have hypergonadotropic hypogonadism
(**A**) Audiogram of affected individual F1 II-4. All three affected sisters show a similar audiometric configuration to F1-II-4, with profound hearing loss across all tested frequencies. (**B**) Audiogram of affected individual F2 II-1. The proband shows bilateral mild to moderate cookie-bite sensorineural hearing loss (SNHL). In both audiograms, the hearing level of the left ear is represented by the blue crosses and the right ear by red circles. The hearing threshold level of a normal adult is 0-20 dB.[1] Audiograms generated using AudGen software. (**C**) Hormone profiles for the three affected sisters in family F1, indicative of hypergonadotropic hypogonadism. Levels of follicle stimulating hormone, luteinising hormone and estrogen in the postmenopausal range are in bold.[2]

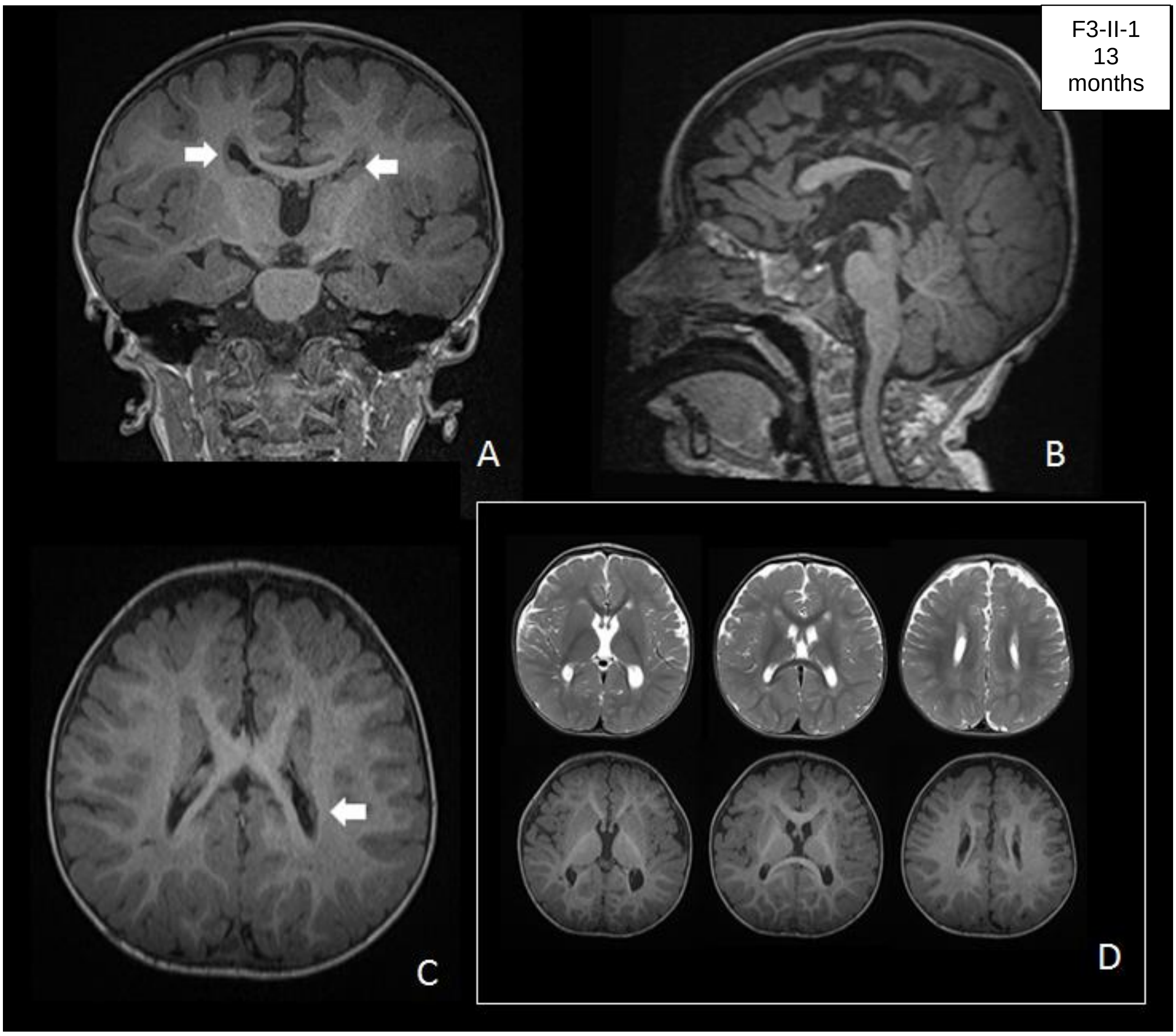
F3-II-1
13
months
A
B
C
D

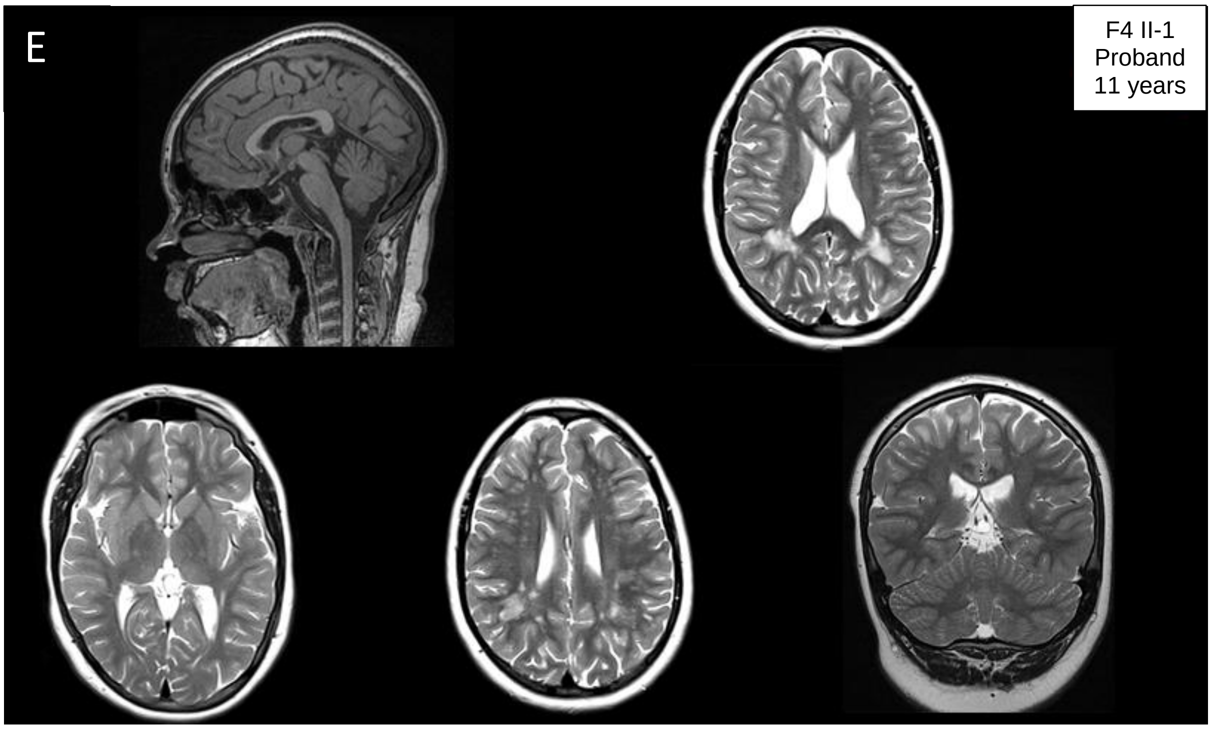
E
F4 II-1
Proband
11 years

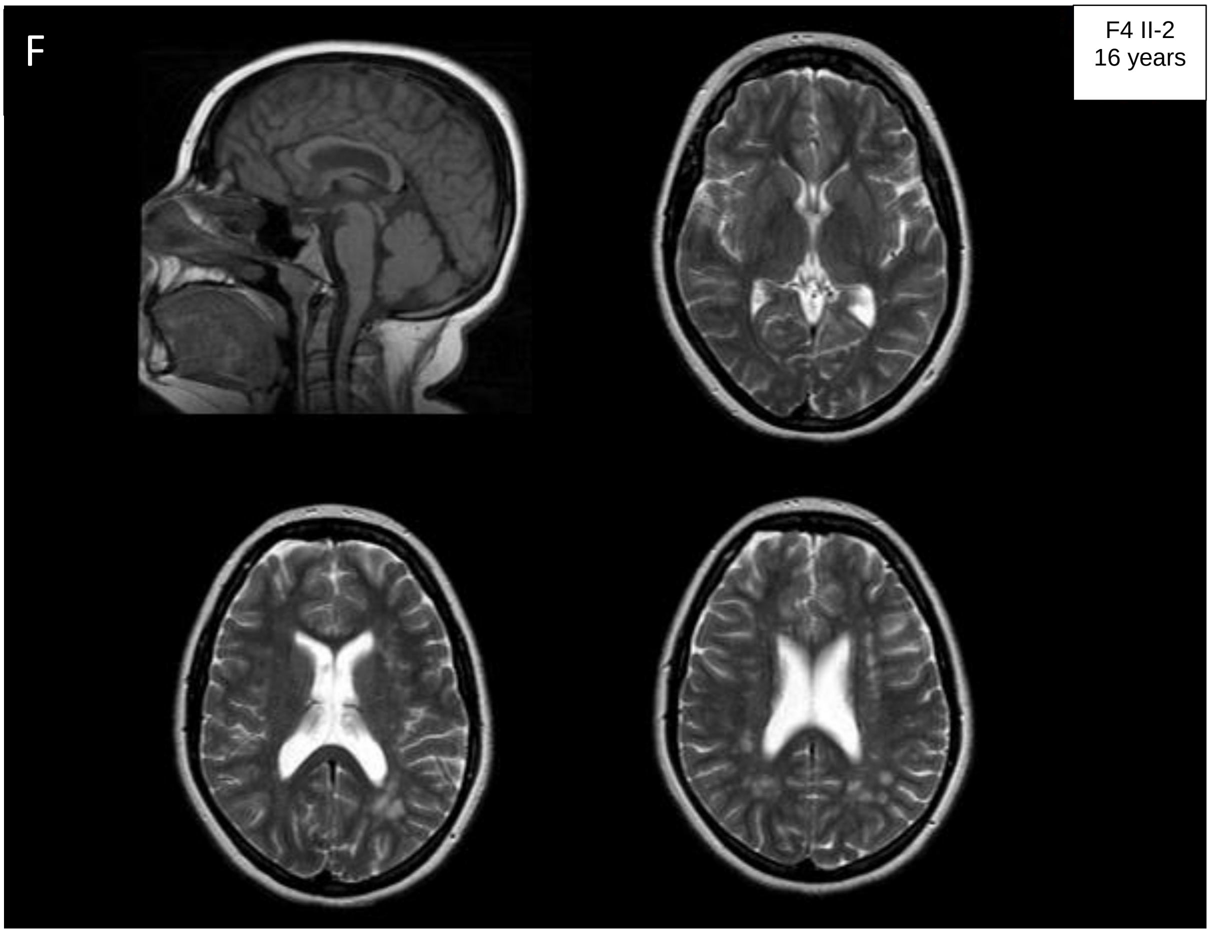
F
F4 II-2
16 years

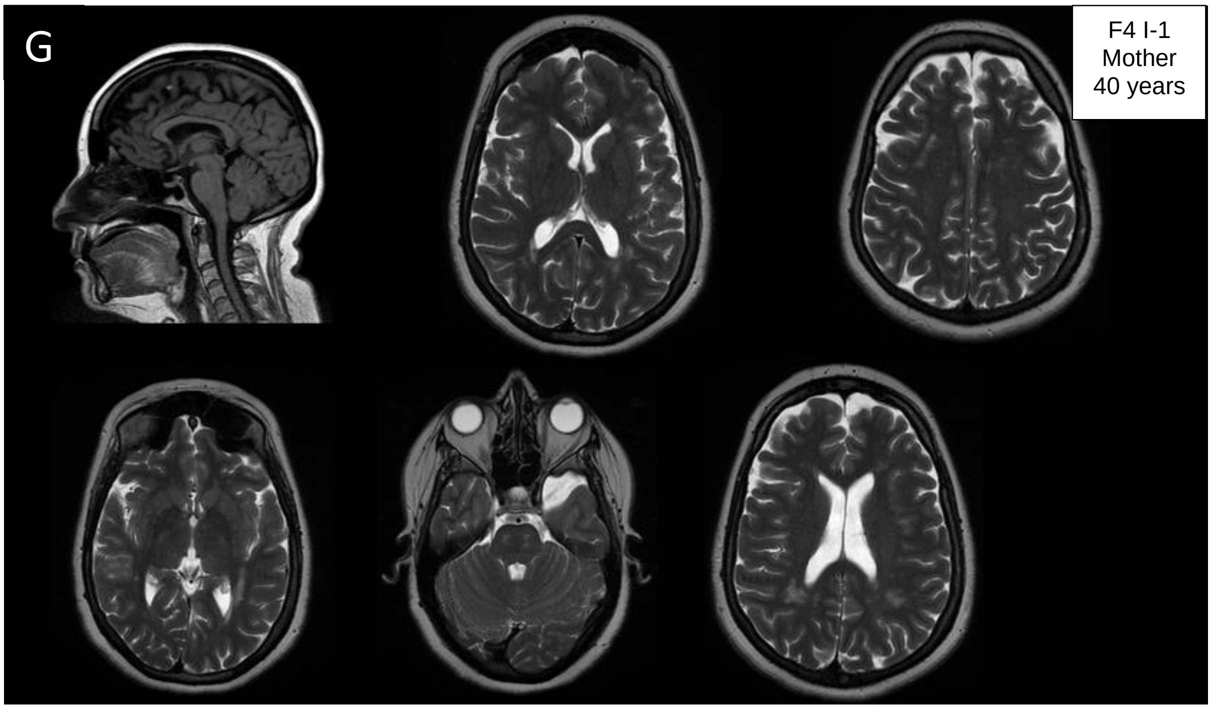

Figure S2 – Brain MRIs for affected individuals in F3 and F4
(A-D) Brain MRI at 13 months of age of individual F3-II-1. Note periventricular cysts – located just above body of lateral ventricles, consistent with connatal cysts (A), dysplastic corpus callosum (B), focal nodular thickening of the posterior horn of left lateral ventricle which may represent a focus of nodular heterotopia (C) and prominent 3rd & lateral ventricles with mild underdevelopment of white matter (D). (F-G) Brain MRI for affected individuals from family F4. Note bilateral multiple periventricular and subcortical T2 white matter hyperintense lesions with a posterior predominance in all affected individuals from this family. The affected mother shows hyper intense lesions involving also the pons (G; bottom right image). For all images age at time of assessment is noted in the white box in the upper right corner of the image.

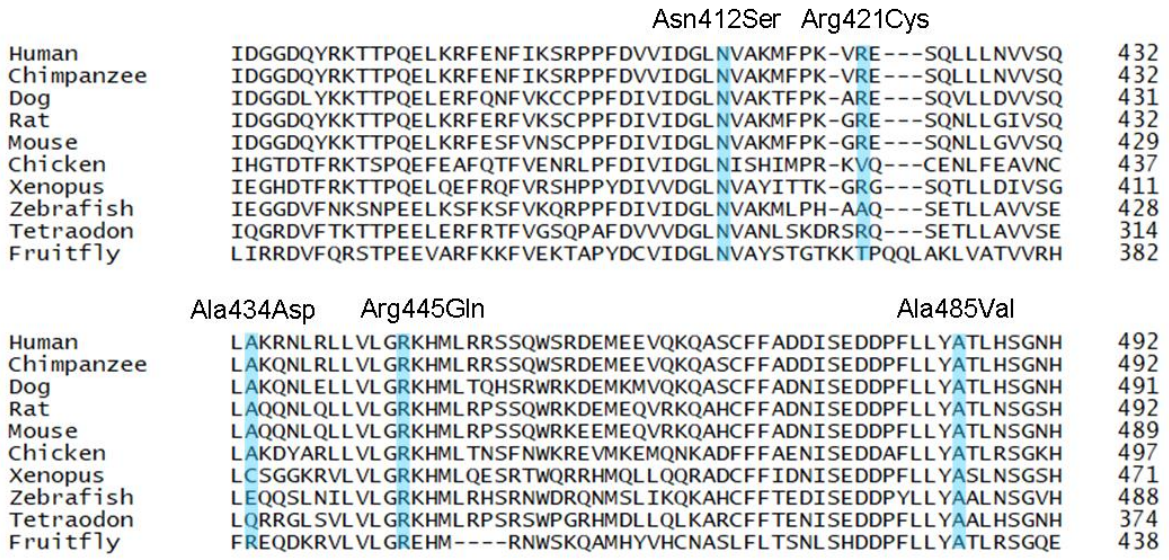

Figure S3 - Conservation of PRORP with variant residues highlighted

The Conservation of PRORP across multiple species. The variant residues in the affected families are highlighted with blue boxes. Numbering relates to the human protein (GenPept: NP_055487.2). The multiple sequence alignments shown use the single letter abbreviations for amino acids while the variants use the three letter HGVS nomenclature. Sequences for each species are as follows; *P. troglodytes* UniProt: H2Q865, *C. lupus familiaris* UniProt: E2RMR1, *R. norvegicus* GenPept: NP_001100200.1, *M. musculus* GenPept: NP_079649.1, *X. tropicalis* UniProt: F6QNJ3, *D.rerio* UniProt: X1WBZ5, *T. nigroviridis* UniProt: H3CE56, *D. melanogaster* GenPept: NP_572309.2.

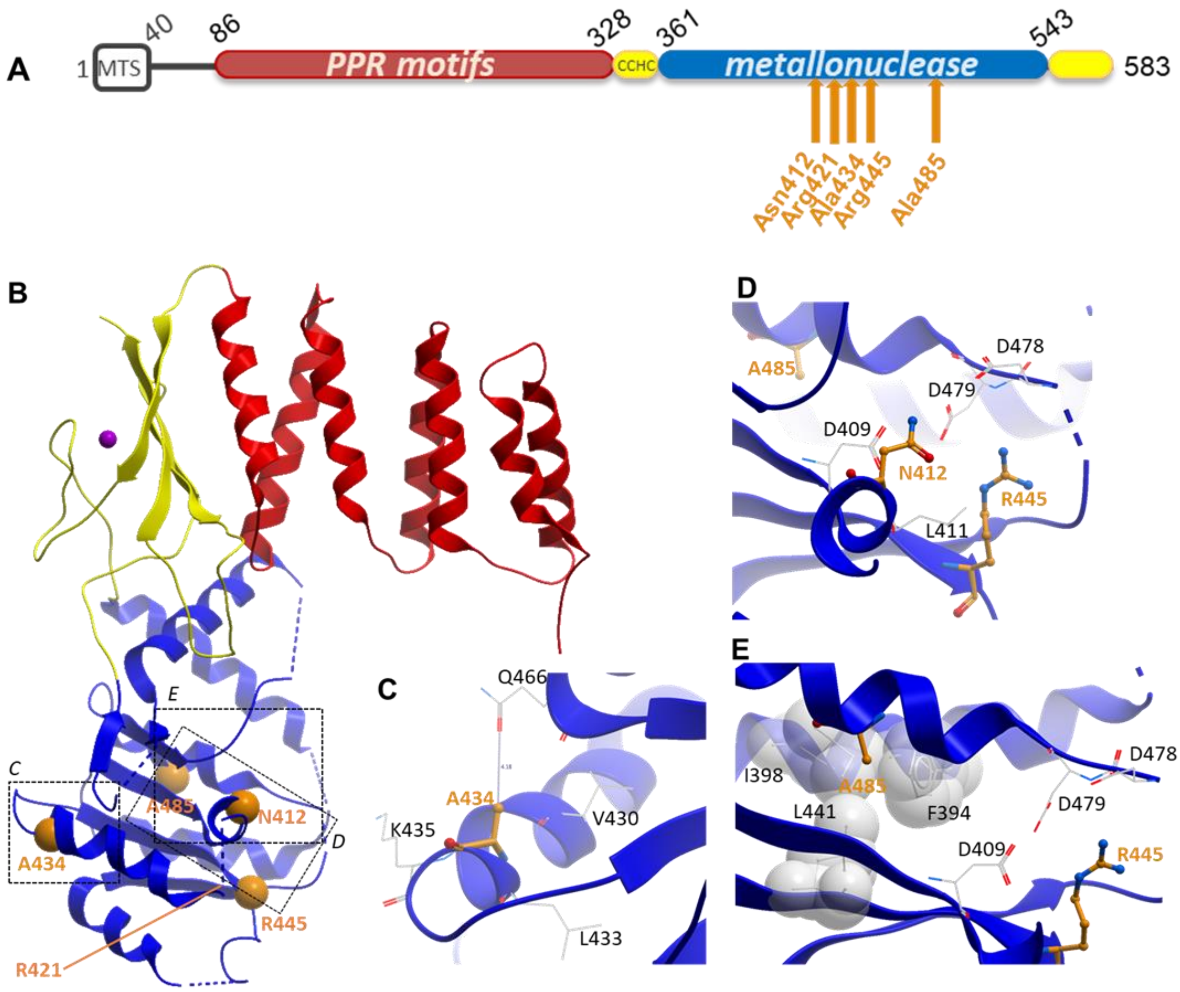

Figure S4 – Localisation of the variant residues in PRORP

(**A**) A schematic domain representation of human PRORP.[3] The location of the variant residues (orange) is noted below the representation. Mitochondrial targeting sequence, MTS; pentatricopeptide repeat domain, PPR. The three letter HGVS nomenclature is used for amino acids (**B**) 3D schematic representation of the protein structure of human PRORP as a ribbon diagram; the enlarged region is part of the metallonuclease domain. (**C, D, E**) The protein structure of human PRORP; the enlarged region is part of the metallonuclease domain. Single letter abbreviations for amino acids are used. The variant amino acids in patients are depicted in orange. Amino acids with interactions with the variant residues are shown in black. The colour of each domain mirrors that of the schematic diagram in A.

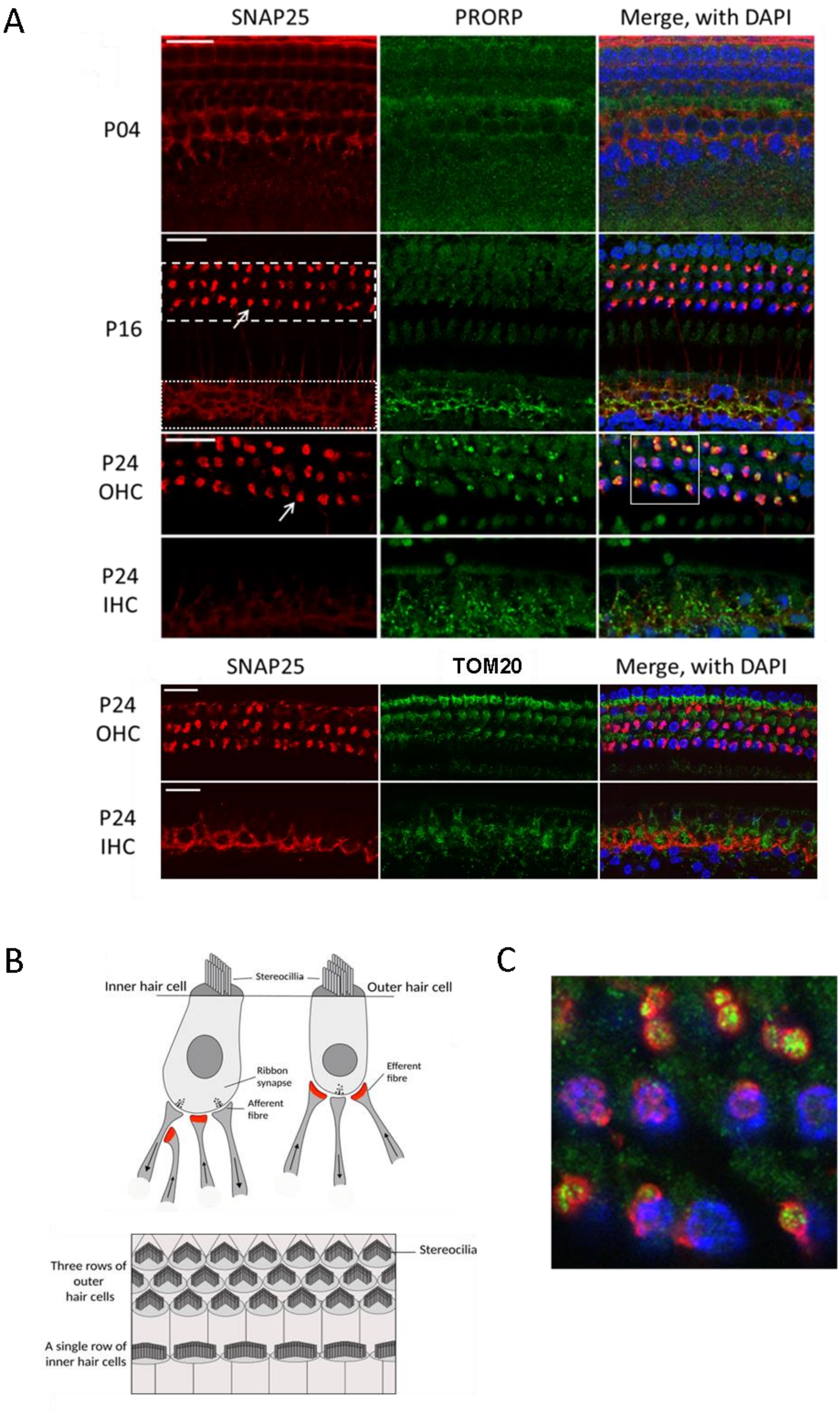
A
SNAP25
PRORP
Merge, with DAPI
P04
P16
P24
OHC
P24
IHC
SNAP25
TOM20
Merge, with DAPI
P24
OHC
P24
IHC
B
Stereocilia
Inner hair cell
Outer hair cell
Ribbon synapse
Efferent fibre
Afferent fibre
Three rows of outer hair cells
A single row of inner hair cells
Stereocilia
C

Figure S5 – Localisation in the mouse organ of Corti reveals high levels of PRORP in the synapses and nerve fibres of hair cells.

(**A**) Confocal fluorescence microscopy optical sections of the whole mount organ of Corti samples from C57/BJ6 mice at postnatal days 4, 16 and 24 (P04, P16 and P24, correspondingly) showing localization of PRORP protein (green). Samples were counterstained with DAPI (nuclear DNA marker, blue) to visualize the nuclei of hair cells and SNAP25 (presynaptic membrane marker, red) to stain efferent synapses at the base of OHCs and nerve fibres and synaptic buttons at the base and around IHCs. Samples at P24 were also stained with TOM20 (mitochondrial marker) as well as SNAP25 and DAPI to show generalized mitochondrial localisation. In the panels for P04, no high level of PRORP immunoreactivity is observed, while at P16, after the onset of hearing, the PRORP signal accumulates in the nerve fibres and at synaptic buttons at the base of IHCs and begins to accumulate at the efferent synaptic buttons of OHCs. The dashed white line outlines the area of the outer hair cell (OHC) efferent synapses, and the dotted white line outlines the area around the inner hair cell (IHC) nuclei and synaptic area. The white arrows point to one of the OHC efferent presynaptic buttons. The P24 panels represent two optical sections through the same organ of Corti sample at different focal plains to visualize OHC synaptic area (top) and IHC synaptic area (bottom). Note that by P24, in the fully mature organ of Corti, the PRORP signal concentrates in both OHC efferent synaptic buttons and in the afferent synaptic buttons of IHCs. The scale bar is 20 µm. (**B**) Schematic representation of the hair cells of the organ of Corti. The top illustration displays the innervation of inner hair cells and outer hair cells during postnatal development. Arrows in the nerve fibres indicate the direction of transmission. The area of SNAP25 staining is shown in red. The lower panel illustration shows the arrangement of hair cells in the organ of Corti in the same orientation as shown in Panel A. (**C)** An enlarged view of the area inside the white box in Panel A showing a concentrated signal of PRORP (green) in the efferent buttons stained by SNAP25 (red); the OHC nuclei shown in blue.

Supplemental Web Resources

AudGen, http://audism.com/audgen

GenBank, https://www.ncbi.nlm.nih.gov/genbank/

UniProt, https://www.uniprot.org/

Materials and Methods

Ethical approval

All individuals or their guardians provided written informed consent in accordance with local regulations. Ethical approval for this study was granted by the National Health Service Ethics Committee (16/WA/0017), University of Manchester; the ethics committee of IDIBELL (CEIC n. PR076/14), and CHEO ethics committee for the Care4Rare Canada Study (ID: 1577), and Genomics England has approval from the HRA Committee East of England – Cambridge South (REC Ref 14/EE/1112).

Autozygosity mapping and whole exome sequencing

Autozygosity mapping was performed on six members of family F1 (II-1, II-2, II-3, II- 4, II-6 and II-7) using the Affymetrix Genome-wide SNP6.0 arrays as previously described.[4] Autozygosity mapping of the array data was performed using the AutoSNPa software.[5] Whole exome sequencing was performed on DNA extracted from lymphocytes from individual F1-II-3. The Agilent SureSelect Human All Exon V5 Panel was used for library preparation and sequencing was performed on the HiSeq 2500 (Illumina) as previously described.[6] Analysis of large deletions and copy number variations was performed on F3-II-1 using Affymetrix Cytoscan HD arrays with analysis via Affymetrix ChAS software (GRCh37/hg19). CNVs were assessed with comparison to databases including the Database of Genomic Variants (DGV). Pathogenic CNVs were identified with reference to databases, including Decipher, ClinVar and ClinGen (ISCA). Whole exome sequencing performed on genomic DNA from all three members of family F3 was performed as a service by GeneDx. Library preparation was performed using the GeneDx propriety technology and sequenced using an Illumina sequencer and using paired-end reads. Reads were aligned to the human genome build GRCh37/hg19 and variants identified using GeneDx software, XomeAnalyzer.

Identification of variants

Autozygosity mapping, performed on six siblings, identified three homozygous regions >2Mb shared between the affected individuals, but not with the unaffected individuals (chromosome 14: 34195478-37228220; chromosome 18: 10090808-12264512; and chromosome 22: 21317876-23416005, Genome build: Hg19). Whole exome sequencing was performed on one affected individual (F1, II-3). After sequence variants in the homozygous regions were filtered to remove variants seen more than once in >800 previously sequenced

exomes, and variants with a minor allele frequency above 1% in dbSNP or the Exome Variant Server (EVS)[7; 8] one variant remained, *PRORP* c.1454C>T; p.(Ala485Val) (Genbank: NM_014672.3).

The variants in family F2 were identified from whole genome sequence data generated through the 100,000 Genomes Project[9] on the parents and affected child and accessed through the dedicated research portal. The trio genome dataset in this family was filtered initially to identify Tier 1 or 2 variants (i.e. rare or known pathogenic variants in genes known to be associated with sensorineural hearing loss). An agnostic approach consistent with an ultra-rare minor allele frequency ($<10^{-5}$) and recessive inheritance pattern revealed maternally inherited *PRORP* c.1235A>G, p.(Asn412Ser) and paternally inherited c.1301C>A, p.(Ala434Asp) variants.

For F3 whole exome sequencing of the family trio was undertaken by GeneDx. Exonic and flanking splice junctions were captured using a proprietary system developed by GeneDx and sequenced on an Illumina platform with 100bp or greater paired end reads. Reads were aligned to human genome build GRCh37/UCSC hg19, and analysed for sequence variants using a custom-developed analysis tool (Xome Analyzer). Mean depth of coverage 94X, quality threshold 98.7%. All available data files were run through the most recent iteration of our previously described bioinformatics pipeline.[10] Family-based reanalysis was completed at the Children's Hospital of Eastern Ontario in collaboration with the referring clinical care team which included (Care4Rare study team members, including the local referring clinician, a clinical geneticist, a laboratory geneticist, genetic counsellor, and/or a post-doctoral fellow). Biallelic variants were identified in *PRORP*, a maternally inherited c.1334G>A p.(Arg445Gln) (rs777185638) and a paternal frameshift variant c.1197dupA p.(Ser400IlefsX6) (rs764714439) were identified.

For F4 genomic DNA was extracted from peripheral blood using standard methods. For WGS, a PCR-free library with 150-bp paired-end read sequences was generated on a HiSeq 2000-4000 platform (Illumina, Inc. USA) at Centre Nacional d'Anàlisi Genòmica (CNAG Barcelona, Spain). Sequences were aligned to hg19 by Burrows-Wheeler Aligner (BWA mem), and single nucleotide variants and small insertions/deletions (indels) were identified using GATK, applying GATK's best practices for germline SNP & indel discovery and annotated by ANNOVAR software. Copy number variants (CNVs) were analyzed by the R package ExomeDepth that uses read-depth data from targeted sequencing experiments and filtered with the Database of Genomic Variants that provides a comprehensive summary of structural variation in the human genome. A recessive model was applied and ultra-rare variants at a minor allele frequency of $<10^{-5}$ and CADD score >20 were filtered. The one candidate variant *PRORP* c.1261C>T, p.Arg421Cys was validated and tested for co-segregation in all family members by Sanger sequencing.

There was no evidence of any other putative disease associated variants in the genome and exome datasets in the four families.

Confirmation of variants

Variants were confirmed in family F1 via Sanger sequencing using the ABI big Dye v3.1 (ThermoFisher) sequencing technology. Primers used were *PRORP*_exon7_ FWD (5'ACACTGTCCTCTGCCTCTTC3') and *PRORP*_ exon7_REV (5'TCTAGGACCTGGCTAGTTCC3')

_PRORP t_ranscript analysis

Dermal fibroblasts from individual F3, II-1 were grown under standard conditions either in the presence of puromycin (200µg/ml) or without puromycin treatment. RNA was extracted using the Qiagen MiniprepRNA kit and RNA samples were DNAse I treated on column. RNA was converted to cDNA using the Applied Biosystems RNA to cDNA kit. Samples were PCR amplified using primers that cross exon-exon borders and Sanger sequenced using ABI big Dye v3.1 (ThermoFisher) sequencing technology. Primers used were *PRORP*_cDNA_Exon 5_Fwd (5'CATGGTTTGAGAGTGTTCCTGG3') and *PRORP*_cDNA_Exon 5 (5'CTGAGAATACGCTGAAAGGTTAG3').

Assessment of protein and RNA levels in fibroblasts

Western blots for the subunits of mt-RNase P, respiratory chain complexes and mitoribosomal proteins (n=3 for each experiment) were performed using fibroblast cell lysates (F1 II-4 and F3 II-1). Cell lysates were incubated with sample dissociation buffer, separated by 12% SDS–PAGE and immobilized by wet transfer on to PVDF membrane (Immobilon-P, Millipore Corporation). Proteins of interest were bound by overnight incubation at 4°C with primary antibodies followed by HRP-conjugated secondary antibodies (Dako Cytomation) and visualized using ECL-prime (GE Healthcare) and BioRad ChemiDoc MP with Image Lab software. Antibodies used are as follows; TRMT10C (Sigma HPA036671), SDR5C1 (Sigma HPA001432), PRORP (Abcam ab185941), SDHA (Abcam ab14715), NDUFB8 (Abcam ab110242), MT-CO1 (ab14705), UQCRC2 (Abcam 14745), ATP5B (Abcam 14730), MT-CO1 (ab14705), and GAPDH (Abcam 8245) followed by HRP-conjugated secondary antibodies (Dako Cytomation).
Northern blot analysis was performed as previously described.[11] The NorthernMax kit from Ambion was used. Equal amounts of total RNA (2–5 µg range) from fibroblasts was separated on a 1% denaturing agarose gel. RNA was then transferred to nylon membrane (Hybond-N + Amersham, GE Healthcare) by capillary transfer, UV cross-linked and subjected to hybridization with biotinylated probes. Signals were detected using the BrightStar BioDetect kit (Ambion). A biotinylated RNA size marker (BrightStar RNA Millenium Marker, Ambion) was used to determine the size of RNA species. Probe sequences as previously described.[11]

Preparation of the PRORP variant sequences for bacterial expression

The plasmid pET28-b(+) containing the coding sequence for PRORP (MRPP3)[12] was mutagenized as previously described[13] with the synthetic oligonucleotides PRORP_p.A485V (5' GGAGTGCAGTGTG**A**CATACAGAAGGAATGG 3'),PRORP_p.R445Q (5'

CGTCTTAGCATGTGCTTC**T**GGCCTAGGACCAGCAGTCG 3'), PRORP_p.N412S (5' GGAAACATTTTGGCAACA**C**TGAGACCATCAATGACAAC 3'), PRORP_p.A434N (5' CAGTCGCAGATTCCGTTTG**T**CTAGTTGAGAGACGACATTC 3') and PRORP_p.R421C (5' CGACATTCAAGAGAAGTTGAGATTCAC**A**AACTTTAGGAAACATTTTGGC 3') with the base altered from wild-type bold and underlined. The potential mutagenized plasmids were extracted using the GenElute HP Plasmid miniprep Kit (Sigma Aldrich) and the variant was confirmed by DNA sequencing.

Recombinant expression and purification of TRMT10C, SDR5C1 and PRORP

PRORP and PRORP variants, as described above, were expressed in *E.coli* Rosetta2 DE3 (Novagen) using Overnight Express TB medium (Novagen). Affinity chromatography of the His-tagged proteins was performed as previously described.[12] Purity was assessed by SDS-PAGE. Aliquots of purified proteins were dialysed overnight at 4°C in 20 mM Tris-Cl pH 7.4, 100 mM NaCl, 15% glycerol, then flash frozen and stored at -80°C. *TRMT10C* in pET28-b(+)[12] was subcloned into pET21d and co-expressed with SDR5C1 in pET28-b(+)[12] in *E.coli* Rosetta2 DE3 using Overnight Express TB medium at 19°C. Purified proteins were dialysed overnight at 4°C in 20 mM Tris-Cl pH 8, 200 mM NaCl, 2mM DTT, 15% glycerol, then flash frozen and stored at -80°C.

Preparation of mitochondrial pre-tRNA transcripts

The template for pre-tRNA^{Ile} (phI2) was as described previously.[12] *In vitro* transcription was carried out with the T7 RiboMax Express system (Promega) according to the manufacturer's instructions with phI2 linearized with XbaI and 2.5µM aminoallyl-UTP-ATTO-680 (Jena Biosciences). RNA was purified by ethanol precipitation.

Pre-tRNA processing assays

Pre-tRNA processing assays were performed as previously described.[12,14] 6% (w/v) acrylamide 8M urea gels were used to resolve ATTO-680 labelled mt-tRNA substrate and cleavage products. Gels were visualised using the LI-COR Odyssey CLx imaging system and band quantitation carried out using the Image Studio software. Aliquots of the tRNA processing reactions were taken at the start of the reaction (0 minutes), after 30 minutes and 60 minutes from the start in three independent assays. Visible processed tRNA bands at time-points 30 minutes and 60 minutes were measured as a proxy for mt-RNase P activity. The relative intensities of tRNA processed by mt-RNase P with wild-type and PRORP variants from three independent assays were quantitated at 30 minutes and presented with standard deviation.

Rescue experiments

RNA isolation and northern blotting

Total RNA from cultured cells was isolated with the Monarch Total RNA Miniprep kit (NEB) according to the manufacturer's instructions. For northern blotting, 5 µg of total RNA

from each sample was separated through a 1.2% agarose-formaldehyde gel and transferred to HybondTM-N+ membrane (GE Healthcare) by neutral transfer. Using T4 Polynucleotide Kinase (NEB) and ATP (γ-^{32}P), an oligonucleotides probe (MT-ATP8, 5’-TGGGTGATGAGGAATAGTGTAAGGAG) was radiolabeled for hybridisation (25% Formamide, 7% SDS, 1% BSA, 0.25M sodium phosphate pH 7.2, 1mM EDTA pH 8.0, 0.25M NaCl2) overnight at 37°C. Membranes were washed first with 2× SSC/0.1% SDS for 60 min, followed by 0.5× SSC/0.1% SDS for 60 min and finally in 0.1× SSC/0.1% SDS for 30 min. All washings were performed at 37°C. The membranes were dried, exposed to a Phosphoscreen (GE Healthcare) and scanned with Typhoon 9400 (GE Healthcare).

Retroviral expression

Full-length cDNAs of human TRMT10C and PRORP were generated by reverse transcription using Superscript IV (Invitrogen) with an oligo dT primer from total RNA isolated from wild-type cultured fibroblasts followed by PCR using specific primers with KAPA HiFi (Sigma-Aldrich) for Gateway cloning into pDONR201. Both cDNAs were verified by Sanger sequencing and sub-cloned into a Gateway-converted pBABE-puromycin retroviral vector. Retrovirus was generated following transfection of plasmids into the Phoenix packaging cell line, followed by transduction into immortalized wild-type and fibroblasts from an affected individual (F3, II-1). Transduced cells were selected with puromycin to select for stable cultures.

Immunoblotting

Cells were lysed in phosphate buffered saline, 1% dodecyl-maltoside (DDM), 1 mM PMSF (phenylmethylsulfonyl fluoride), and complete protease inhibitor (Thermo Fisher Scientific). Protein concentration of lysates was measured by the Bradford protein assay (BioRad) and equal amounts separated in 12% Tris-Glycine SDS-PAGE. Proteins were transferred to nitrocellulose membranes by semi-dry transfer. Membranes were blocked in TBST (Tris-buffered saline, 0.1% Tween 20) with 1% milk at room temperature for 1 hr. Primary antibodies (in 5% BSA/TBST) were incubated overnight at 4°C and detected the following day with secondary HRP conjugates (Jackson ImmunoResearch) using ECL (LumiGLO, Cell Signalling Technology) with film. The following primary antibodies were used for immunoblotting: Proteintech Group: MRPP3 (20959-1-AP, 1:3000); Abcam/Mitosciences: MT-CO1 (1D6E1A8, 1:500) and SDHA (C2061/ab14715, 1:10000); Santa Cruz: TOM40 (sc-11414, 1:5000) and Thermo Fisher Scientific: MRPP1 (A304-390A, 1:1000).

Immunohistochemistry

The NIH Animal Care and Use Committee approved protocol 1263-15 to T.B.F. for mice. C57/BJ6 mice at ages P04, P16 and P24 were euthanised, the cochleae were removed and fixed with 4% paraformaldehyde in PBS for 2 hours. The samples were microdissected and the organ of Corti was permeabilised with 0.5% Triton X-100 in PBS for 30 min followed by three 10 min washes with 1X PBS. Nonspecific binding sites were blocked with 5% normal goat serum and 2% BSA in PBS for 1 h at room temperature. Samples were incubated for 2 h

with rabbit polyclonal PRORP antibody (MRPP3, Proteintech, 20959-1-AP) at 1µg/ml and mouse monoclonal SNAP25 antibody (Santa Cruz, sc-136267) at 1µg/ml or rabbit polyclonal Tom20 antibody (Santa Cruz, sc-11415) at 2µg/ml and mouse monoclonal SNAP25 (Santa Cruz, sc-136267) at 1µg/ml followed by several rinses with PBS. Samples were incubated with goat anti-rabbit IgG Alexa Fluor 488 conjugated secondary antibody and goat anti mouse Alexa Fluor 568 conjugated secondary antibody (Molecular Probes) for 30 min. Samples were washed several times with PBS, with ProLongGold Antifade reagent with DAPI (Molecular Probes) and examined using an LSM780 confocal microscope (Zeiss Inc) equipped with 63X, 1.4 N.A. objective.

Acknowledgements

This study was supported by Action on Hearing Loss (S35); Action Medical Research (GN2494); NIHR Manchester Biomedical Research Centre ((IS-BRC-1215-20007); Wellcome Trust ISSF pump-prime award (097820/Z/11/B); the Wellcome Trust Centre for Mitochondrial Research (203105/Z/16/Z to RWT); the UK NHS Highly Specialised “Rare Mitochondrial Disorders of Adults and Children” Service (RWT); and The Lily Foundation (RWT); Austrian Science Fund (FWF) P25983 (WR); in part by the Intramural Research Program of the NIDCD at the NIH, (DC000039 to TBF); the Sigrid Juselius Foundation Senior Investigator Award (BJB; grants from the Hesperia Foundation; the Asociación Española contra las Leucodistrofias (ALE-ELA España), the PERIS program URD-Cat SLT002/16/00174); the Center for Biomedical Research on Rare Diseases (CIBERER) (ACCI19-759 to A.P); Fundació La Marató de TV3 (595/C/2020); Instituto de Salud Carlos III (FIS PI20/00758) (co-funded by European Regional Development Fund. ERDF, a way to build Europe); the Instituto de Salud Carlos III (Sara Borrell, CD19/00221 to E.V.), co-funded by European Social Fund; ESF investing in your future, and the Ministerio de Ciencia e Innovación y Universidades (Juan de la Cierva, FJCI-2016-28811 to E.V.). We also thank the CERCA Program/Generalitat de Catalunya for institutional support. This research was made possible through access to the data and findings generated by the 100,000 Genomes Project. The 100,000 Genomes Project is managed by Genomics England Limited (a wholly owned company of the Department of Health and Social Care). The 100,000 Genomes Project is funded by the National Institute for Health Research and NHS England. The Wellcome Trust, Cancer Research UK and the Medical Research Council have also funded research infrastructure. The 100,000 Genomes Project uses data provided by patients and collected by the National Health Service as part of their care and support. Thanks to Christie Boswell-Patterson for providing information from the Care4Rare Study. This work was performed under the Care4Rare Canada Consortium funded by Genome Canada and the Ontario Genomics Institute (OGI-147), the Canadian Institutes of Health Research, Ontario Research Fund, Genome Alberta, Genome British Columbia, Genome Quebec, and Children’s Hospital of Eastern Ontario Foundation.

References

1. Action on Hearing Loss. (2017). Levels of hearing loss. In. (Action on hearing loss.
2. Aiman, J., and Smentek, C. (1985). Premature ovarian failure. Obstet Gynecol 66, 9-14.
3. Reinhard, L., Sridhara, S., and Hallberg, B.M. (2015). Structure of the nuclease subunit of human mitochondrial RNase P. Nucleic Acids Res 43, 5664-5672.
4. Banka, S., Blom, H.J., Walter, J., Aziz, M., Urquhart, J., Clouthier, C.M., Rice, G.I., de Brouwer, A.P., Hilton, E., Vassallo, G., et al. (2011). Identification and characterization of an inborn error of metabolism caused by dihydrofolate reductase deficiency. Am J Hum Genet 88, 216-225.
5. Carr, I.M., Flintoff, K.J., Taylor, G.R., Markham, A.F., and Bonthron, D.T. (2006). Interactive visual analysis of SNP data for rapid autozygosity mapping in consanguineous families. Hum Mutat 27, 1041-1046.
6. Smith, M.J., Beetz, C., Williams, S.G., Bhaskar, S.S., O'Sullivan, J., Anderson, B., Daly, S.B., Urquhart, J.E., Bholah, Z., Oudit, D., et al. (2014). Germline mutations in SUFU cause Gorlin syndrome- associated childhood medulloblastoma and redefine the risk associated with PTCH1 mutations. J Clin Oncol 32, 4155-4161.
7. Sherry, S.T., Ward, M.H., Kholodov, M., Baker, J., Phan, L., Smigielski, E.M., and Sirotkin, K. (2001). dbSNP: the NCBI database of genetic variation. Nucleic Acids Res 29, 308-311.
8. NHLBI GO Exome Sequencing Project (ESP). Exome Variant Server. In. (Seattle, WA).
9. Turnbull, C., Scott, R.H., Thomas, E., Jones, L., Murugaesu, N., Pretty, F.B., Halai, D., Baple, E., Craig, C., Hamblin, A., et al. (2018). The 100 000 Genomes Project: bringing whole genome sequencing to the NHS. BMJ 361, k1687.
10. Kernohan, K.D, Hartley, T., Alirezaie, N., Care4Rare Canada Consortium, Robinson, P.N., Dyment, D.A., Boycott, K.M. (2018). Evaluation of exome filtering techniques for the analysis of clinically relevant genes. Hum Mutat 39:197-201.
11. Deutschmann, A.J., Amberger, A., Zavadil, C., Steinbeisser, H., Mayr, J.A., Feichtinger, R.G., Oerum, S., Yue, W.W., and Zschocke, J. (2014). Mutation or knock-down of 17beta-hydroxysteroid dehydrogenase type 10 cause loss of MRPP1 and impaired processing of mitochondrial heavy strand transcripts. Hum Mol Genet 23, 3618-3628.
12. Holzmann, J., Frank, P., Loffler, E., Bennett, K.L., Gerner, C., and Rossmanith, W. (2008). RNase P without RNA: identification and functional reconstitution of the human mitochondrial tRNA processing enzyme. Cell 135, 462-474.
13. Kunkel, T.A., Roberts, J.D., and Zakour, R.A. (1987). Rapid and efficient site-specific mutagenesis without phenotypic selection. Methods Enzymol 154, 367-382.
14. Rossmanith, W., Tullo, A., Potuschak, T., Karwan, R., and Sbisa, E. (1995). Human mitochondrial tRNA processing. J Biol Chem 270, 12885-12891.
